# Cardiac Performance in the First Year of Age Among Preterm Infants Fed Maternal Breast Milk

**DOI:** 10.1001/jamanetworkopen.2021.21206

**Published:** 2021-08-27

**Authors:** Afif El-Khuffash, Adam J. Lewandowski, Amish Jain, Aaron Hamvas, Gautam K. Singh, Philip T. Levy

**Affiliations:** 1 Department of Neonatology, The Rotunda Hospital and School of Medicine (Pediatrics), Royal College of Surgeons in Ireland, Dublin, Ireland; 2 Oxford Cardiovascular Clinical Research Facility, Division of Cardiovascular Medicine, Radcliffe, Department of Medicine, University of Oxford, Oxford, United Kingdom; 3 Department of Paediatrics, University of Toronto and Lunenfeld-Tanenbaum Research Institute, Mount Sinai Hospital, Toronto, Ontario, Canada; 4 Department of Pediatrics, Northwestern University Feinberg School of Medicine, Chicago, Illinois; 5 Department of Pediatrics, Washington University School of Medicine, Saint Louis, Missouri; 6 Department of Pediatrics, Central Michigan University School of Medicine, Children's Hospital of Michigan, Detroit; 7 Department of Pediatrics, Harvard Medical School, Boston, Massachusetts; 8 Division of Newborn Medicine, Boston Children’s Hospital, Boston, Massachusetts

## Abstract

**Importance:**

There is a beneficial association between human breast milk exposure in the neonatal period and cardiac mechanics in adults who were born preterm. It is unknown whether this benefit is apparent in infants in the first year of age.

**Objective:**

To test the hypothesis that higher consumption of mother’s own milk in preterm infants is associated with enhanced cardiac performance during the first year of age.

**Design, Setting, and Participants:**

This cross-sectional study of cardiac and nutritional data at an academic medical center included 80 individuals born preterm and 100 individuals in the control group born full-term. All births were between 2011 and 2013. Two-dimensional echocardiograms were performed at 32 weeks’ and 36 weeks’ postmenstrual age and at 1 year’s corrected age in individuals born preterm and at 1 month and 1 year of age in individuals born full-term. Statistical analysis was performed from January to May 2021.

**Exposures:**

Consumption of mother’s own milk.

**Main Outcomes and Measures:**

Main study outcomes included echocardiography measures of right and left ventricle longitudinal strain (function), left ventricle mass index and right ventricular areas (morphology), and pulmonary vascular resistance (pulmonary hemodynamics) at age 1 year.

**Results:**

Of 180 infants included in the study, 97 (54%) were Black infants and 89 (49%) were female infants. Among the 80 infants born in the preterm cohort, 43 (54%) were female infants and 43 (54%) were Black infants. The median gestational age at birth of the preterm infants was 27.0 weeks (interquartile range, 26.0-28.0 weeks) and the median birth weight was 960 g (interquartile range, 800–1138). For each week of exposure to mother’s own milk, preterm infants had greater magnitudes of right ventricular strain (eg, right longitudinal strain: β, 0.021; 95% CI, 0.002-0.041; *P* < .001) and left ventricular strain (eg, left longitudinal strain: β, 0.065; 95% CI, 0.049-0.080; *P* = .01), larger right ventricle areas (eg, systolic area: β, 0.026; 95% CI, 0.011-0.042; *P* = .009), larger left ventricle mass index (β, 0.045; 95% CI, 0.024-0.073; *P* = .003), and decreased pulmonary vascular resistance (eg, pulmonary artery acceleration time: β, 0.041; 95% CI, 0.018-0.063; *P* < .001) at 1 year’s corrected age, even after accounting for gestational age and common neonatal morbidities. Cardiac values approached those seen in controls born full-term with increased mother’s own milk exposure. There were no differences in any of the cardiac indices at 32 weeks’ postmenstrual age, but with each week of exposure, right ventricle function (eg, right longitudinal strain: β, 0.016; 95% CI, 0.002-0.031; *P* < .001) was greater and pulmonary pressured (eg, pulmonary artery acceleration time: β, 0.0032; 95% CI, 0.0013-0.0062; *P* < .001).

**Conclusions and Relevance:**

This study found that preterm infants with higher consumption of mother’s own milk had enhanced cardiac performance at age 1 year, suggesting that mother’s own milk consumption may play a dynamic modulator role on cardiac mechanics in preterm-born infants and help in normalization of the preterm cardiac phenotype.

## Key Points

**Question:**

What are the associations of exposure to mother’s own milk on cardiac performance during the first year of age in preterm infants?

**Findings:**

This cross-sectional study of 80 preterm infants found that those with higher exposure to mother’s own milk had enhanced cardiac function and morphology on echocardiographic examination at age 1 year, with values approaching those of full-term infants in the control group.

**Meaning:**

These findings suggest that there is a favorable association between consumption of maternal breast milk and cardiac performance at age 1 year in preterm infants.

## Teaser

This cross-sectional study investigates the association between consumption of maternal breast milk and cardiac performance at age 1 year among individuals born preterm.

## Introduction

Children and adults who were born preterm are at an increased risk of cardiometabolic disorders, including ischemic heart disease, heart failure, systemic and pulmonary hypertension*;* and have higher mortality from cardiovascular disease.^1,2,3,4^ The hearts of young adults who were born preterm demonstrate a unique cardiac phenotype with reduced biventricular volume, shorter length, lower systolic and diastolic function, and a disproportionate increase in muscle mass.^5,6,7^ Exclusive human milk feeding through the first months of postnatal development is associated with normalization of aspects of the preterm cardiac phenotype in young adulthood toward that of those born full-term, suggesting a potential long-term cardioprotective effect of human milk over formula diet during initial hospitalization.^8,9^

Cardiac mechanics begin to undergo maturational changes during the early neonatal period with preterm infants exhibiting impaired right ventricular (RV) performance, persistent pulmonary vascular disease (PVD) and alterations in left and right heart structure that persist to age 1 year.^10^ Bronchopulmonary dysplasia (BPD) and late onset pulmonary hypertension (PH) leave a further negative impact of cardiac performance over the same time period.^11,12^ Although this postnatal developmental window provides an early opportunity for interventions to modulate the long-term cardiac phenotypes,^8^ to our knowledge, the association of breast milk with cardiovascular performance over the first year of age has not been described. Accordingly, we hypothesized that mother’s own milk (MoM) exposure is a modifiable factor that will enhance cardiac performance and pulmonary hemodynamics in this high-risk preterm population by 1 year’s corrected age (CA). The objective of the study was to assess the association between exposure to MoM and cardiopulmonary performance at 1 year’s CA in preterm infants.

## Methods

### Participants

**Figure 1.**
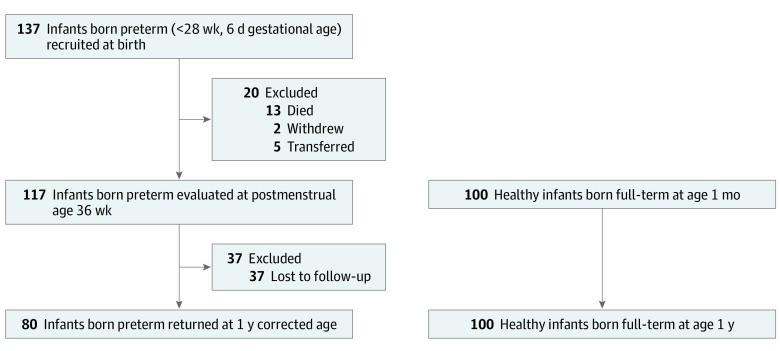
Study Population

In this cross-sectional study, we performed a post hoc analysis of data acquired from preterm infants enrolled through the Prematurity and Respiratory Outcomes Program (PROP) (NCT01435187) site at Washington University School of Medicine and Saint Louis Children’s Hospital between August 2011 and November 2013. Details of the PROP study design have been described elsewhere.^13^ Briefly, 724 infants born between 23 weeks, 0 days and 28 weeks, 6 days of gestation were recruited from 6 academic centers (13 hospitals) in the United States at birth and observed to 1 year’s CA. Infants’ demographic and clinical characteristics were obtained at each site to identify early risk factors of later respiratory morbidity.^14^ At the Washington University PROP site only, infants received echocardiograms at 32 weeks’ postmenstrual age (PMA), 36 weeks’ PMA, and 1 year’s CA. For this current study, 80 participants who received echocardiograms at all 3 time points from the Washington University PROP site were eligible for inclusion (Figure 1). Exclusion criteria are outlined in the eAppendix of the Supplement. A cohort of 100 healthy, age- and sex-matched individuals who were born full-term was recruited for the control group. Race/ethnicity data were collected for descriptive purposes and were investigator acquired and observed. The Washington University institutional review board for human studies at Washington University approved this study. Written informed consents were obtained from the parents and guardians of the study participants. This study followed the Strengthening the Reporting of Observational Studies in Epidemiology (STROBE) reporting guideline for cross-sectional studies.

### Anthropometry, Nutritional, and Clinical Characteristics

For the preterm cohort, the collected anthropometric data included daily weights and weekly lengths. Nutritional information encompassed daily milk consumption of MoM, donor human milk (DHM), and/or bovine formula. Recognizing that infants may have received mixed feedings during a given day, we defined a day of MoM as consumption of MoM greater than 50% of the total volume of enteral nutrition. The following common neonatal outcomes were evaluated: BPD,^15^ late PH^12^ and necrotizing enterocolitis (Bell stage II, with radiological evidence of pneumatosis) (see eAppendix in the Supplement).

### Cardiovascular Echocardiography Acquisition and Analysis

A standardized validated protocol based on the guidelines of the American Society of Echocardiography was used to perform and analyze echocardiograms at 32 weeks’ PMA, 36 weeks’ PMA, and 1 year’s CA in infants born preterm and at 1 month and 1 year of age in infants born full-term.^16,17^ Heart rate, blood pressure readings, the mode of respiratory support, and oxygen requirements were recorded at the time of each echocardiogram. Image data were acquired and stored in raw Digital Imaging and Communications in Medicine cineloop format for offline analysis.

### Echocardiography Measures

Echocardiographic measurements for left ventricular (LV) and RV functional and morphometric parameters, and estimates of pulmonary hemodynamics are described in the eAppendix in the Supplement and outlined as follows: LV function was analyzed by the quantification of 2-dimensional speckle-tracking–derived LV longitudinal strain and strain rate.^18^ LV morphology was assessed with LV mass indexed (LVMi) and relative wall thickness (RWT).^19^ RV function was assessed with fractional area of change (FAC),^20^ tricuspid annular plane systolic excursion (TAPSE),^21^ and RV longitudinal strain and strain rate.^12^ RV morphology was characterized by RV areas in systole and diastole and RV linear dimensions.^20^ RV afterload (pulmonary vascular resistance and compliance) was quantified by assessing pulmonary artery systolic pressure estimated by pulmonary artery acceleration time (PAAT) and ratio of RV ejection time to PAAT (PAATi).^22^ The coupling relationship of RV performance to RV afterload was described by TAPSE/PAATi.^23^

### Statistical Analysis

All demographic data were expressed as median with interquartile ranges (IQRs) or as percentages, and all echocardiographic data were expressed as mean with standard deviation. Continuous variables were tested for normality using the Kolmogorov-Smirnov test and a visual histogram illustration of the data. We used a 2-way mixed analysis of variance (ANOVA) with repeated measures to understand if there was an interaction between the MoM exposure groups and time on cardiac performance. Receiver operating characteristic (ROC) curves were constructed to determine cutoff values for days of MoM exposure to detect RV or LV dysfunction, or elevated RV afterload at 1 year’s CA, defined as magnitude of RV strain less than –27.2%, LV strain less than –20.2%, or PAATi greater than 3, respectively.^11,12,22^ Univariate analysis was used to determine the best risk factors to enter in the model and then backward stepwise regression was performed to assess the independent association of gestational age, sex, race, ethnicity, total oxygen days, length of stay, and common neonatal morbidities known to impact cardiopulmonary development (eg, BPD and late PH),^11,12,20^ while adjusting for weight and body surface area at each examination. Sample size estimates are presented in the eAppendix in the Supplement. *P* < .05 was considered statistically significant. Statistical analyses were performed using SPSS statistical software version 27 (IBM Corp) from January to May 2021.

## Results

### Characteristics of Cohorts

Among the 80 infants born in the preterm cohort, 43 (54%) were female infants and 43 (54%) were Black infants. The median gestational age at birth of the preterm infants was 27.0 weeks (IQR, 26.0-28.0 weeks) and the median birth weight was 960 g (IQR, 800–1138). Maternal and infant clinical and demographic characteristics are presented in Table 1.

**Table 1.** Characteristics of Preterm Cohorts

Characteristic	Infants, No. (%)
Birth weight, median (IQR), g	960 (800-1138)
Gestational age, median (IQR), wk	27 (26-28)
Sex	
Female	43 (54)
Male	37 (46)
Race	
White	37 (47)
Black	43 (54)
Ethnicity	
Hispanic or Latino	1 (1)
Not Hispanic or Latino	79 (99)
Antenatal corticosteroids	66 (83)
Surfactant replacement therapy	80 (100)
Cesarean birth	53 (66)
Maternal complications	
Gestational diabetes	10 (13)
Prolonged rupture of membranes	13 (16)
Chorioamnionitis	8 (10)
Preeclampsia	32 (40)
Nutritional status, median (IQR)	
Mother’s own milk, wk	4 (2-8)
Weight, g	
32 wk PMA	1407 (1200-593)
36 wk PMA	2165 (1785-2511)
1 y	9800 (9100-11 275)
Length at 1 y, cm	72 (66-79)
Postnatal complications	
Bronchopulmonary dysplasia^a^	49 (61)
Pulmonary hypertension at 36 wk PMA^b^	12 (15)
Presence of PDA at 36 wk PMA	8 (9)
Necrotizing enterocolitis (Bell stage II or higher)	12 (15)
Retinopathy of prematurity threshold (stage 2 or higher)	29 (36)
Intraventricular hemorrhage (grade 3 or 4)	16 (20)
Total oxygen time, median (IQR), d	76 (33-100)
Length of stay, median (IQR), d	94 (78-112)

Abbreviations: IQR, interquartile range; PDA, patent ductus arteriosus; PMA, postmenstrual age.

^a^ Bronchopulmonary dysplasia is defined by a modified National Institutes of Health definition (see eAppendix in the Supplement).

^b^ Pulmonary hypertension at 36 weeks is defined by a broad-based echocardiography definition (see eAppendix in the Supplement).

### LV Performance

**Figure 2.**
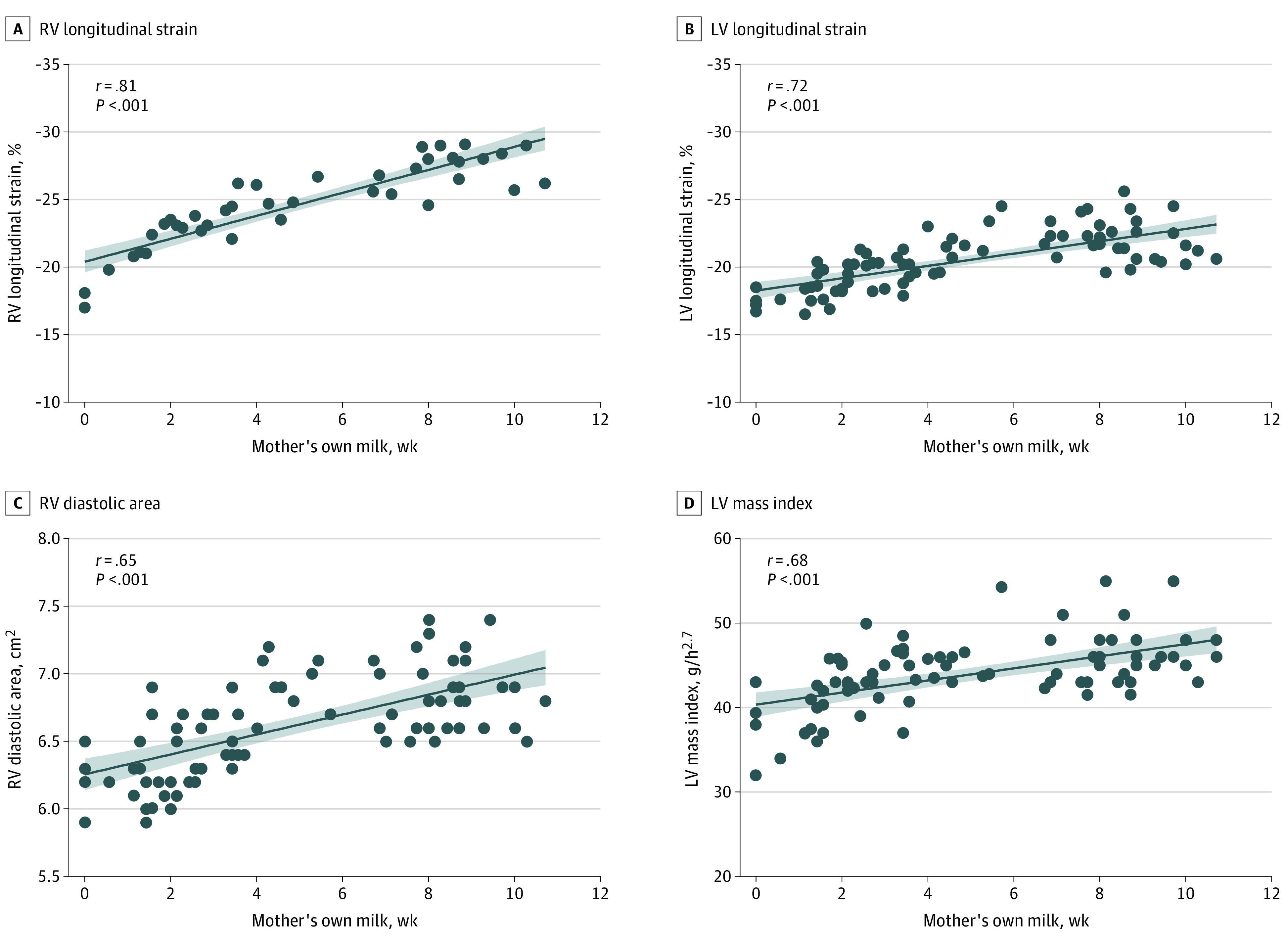
Linear Regression Analysis at Age 1 Year

**Figure 3.**
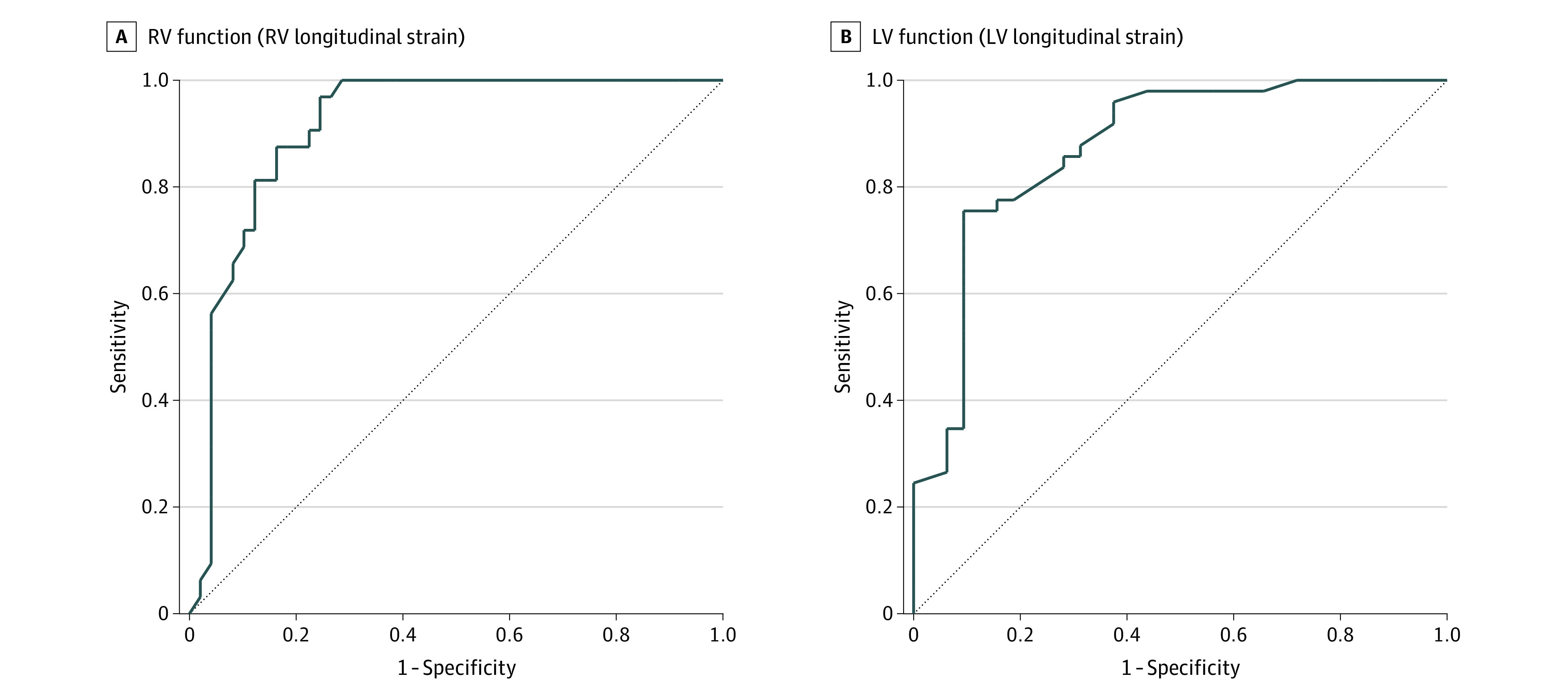
Receiver Operator Curves (ROC) Analysis to Determine Specific Cutoff Values of Days of Mother’s Own Milk in Estimating Left Ventricular (LV) or Right Ventricular (RV) Dysfunction A, For detection of RV dysfunction (RV strain less than –27.7%) at 1 year’s corrected age, a cutoff of less than 27 days of mother’s own milk exposure had a sensitivity of 84% and specificity of 84% with an area under the ROC curve of 0.912 (95% CI, 0.851 to 0.973). B, For detection of LV dysfunction (LV strain less than −20.2%) at 1 year’s corrected age, a cutoff of less than 26 days of mother’s own milk exposure had a sensitivity of 78% and specificity of 81% with an area under the ROC curve of 0.870 (95% CI, 0.793 to 0.961).

At 1 year’s CA, multivariate analysis demonstrated that for each additional week of MoM exposure there was enhanced LV function, as measured by LV longitudinal strain (β, 0.065; 95% CI, 0.049 to 0.080; *P* = .01), larger LVMi (β, 0.045; 95% CI, 0.024 to 0.073; *P* = .003), and decreased RWT (β, −0.052: 95% CI, (−0.092 to −0.011; *P* < .001) even after adjustments for gestational age at birth, sex, heart rate, and common comorbidities (BPD, late PH, and necrotizing enterocolitis) (Table 2). Linear regression analysis demonstrated positive correlation between LV strain and LVMi at 1 year CA and weeks of MoM exposure (Figure 2). For detection of LV dysfunction at 1 year CA, a cutoff less than 26 days of MoM was found to have an area under the ROC curve (AUC) of 0.870 (95% CI, 0.793-0.961) (Figure 3). Compared with full-term infants at age 1 year, LV strain was larger and LVMi was smaller in the preterm infants (eTable 1 in the Supplement). There was no difference in measures of LV function and LV morphology at 32 weeks’ PMA (eTable 2 and eTable 3 in the Supplement), or 36 weeks’ PMA based on the exposure of MoM (eFigure 4 in the Supplement).

**Table 2.** Multivariate Analysis of Cardiac Parameters at Age 1 Year

Cardiac variable	β (95% CI)^a^	*P* value
Left ventricle function		
Longitudinal strain, %	0.065 (0.049 to 0.080)	.01
Longitudinal systolic strain rate, 1/s	0.072 (0.029 to 0.113)	.03
Longitudinal early diastolic strain rate, 1/s	0.051 (0.018 to 0.081)	.02
Longitudinal late diastolic strain rate, 1/s	0.086 (0.032 to 0.107)	.03
Left ventricle morphology		
LV mass index^b^	0.045 (0.024 to 0.073)	.003
Relative wall thickness^c^	–0.052 (–0.092 to –0.011)	<.001
Right ventricle function		
Fractional area of change, %	0.034 (0.010 to 0.075)	.008
Tricuspid annular plane systolic excursion, mm	0.026 (0.013 to 0.053)	.009
Longitudinal strain, %	0.021 (0.002 to 0.041)	<.001
Longitudinal systolic strain rate, 1/s	0.053 (0.022 to 0.097)	<.001
Longitudinal early diastolic strain rate, 1/s	0.065 (0.028 to 0.081)	<.001
Longitudinal late diastolic strain rate, 1/s	0.033 (0.018 to 0.079)	<.001
Right ventricle morphology		
Basal length, cm	–0.032 (–0.082 to –0.012)	.002
Midcavity length, cm	–0.041 (–0.094 to –0.016)	.005
Major length, cm	0.086 (–0.312 to 0.345)	.45
Systolic area, cm^2^	0.026 (0.011 to 0.042)	.009
Diastolic area, cm^2^	0.019 (0.005 to 0.033)	.01
Right ventricle afterload		
PAAT, ms	0.041 (0.018 to 0.063)	<.001
PAATi (RVET/PAAT)	–0.024 (–0.044 to –0.012)	<.001
Right ventricle coupling		
TAPSE/PAATi	–0.018 (0.034 to –0.003)	<.001

Abbreviations: LV, left ventricle; PAAT, pulmonary artery acceleration time; PAATi, indexed pulmonary artery acceleration time; RVET, right ventricle ejection time; TAPSE, tricuspid annular plane systolic excursion.

^a^ Multivariate analyses reported are adjusted absolute differences in means that correspond to a 1 week increase in mother’s own breast milk exposure. Comparisons adjusted for age, sex, bronchopulmonary dysplasia, late pulmonary hypertension, and necrotizing enterocolitis.

^b^ LV mass index is calculated as weight in grams divided by height in meters to the power of 2.7.

^c^ Relative wall thickness = [2 × posterior wall thickness] / LV end diastolic dimensions.

### RV Performance

At 1 year’s CA, multivariate analysis found that for each additional week of MoM exposure there was enhanced RV function, as measured by TAPSE (β, 0.026; 95% CI, 0.013 to 0.053; *P* = .009), FAC (β, 0.034: 95% CI, 0.010 to 0.075; *P* = .003), and RV strain (β, 0.021; 95% CI, 0.002 to 0.041; *P* < .001), larger RV cavity dimensions (eg, basal length: β, −0.032; 95% CI, −0.082 to −0.012; *P* = .002), and larger systolic (β, 0.026: 95% CI, 0.011 to 0.042; *P* = .009) and diastolic areas (β, 0.019: 95% CI, 0.005 to 0.033; *P* = .01), even after accounting for gestational age BPD, late PH, and necrotizing enterocolitis (Table 2). Linear regression analysis demonstrated positive association between measures of RV performance (RV strain and RV diastolic area) at 1 year’s CA and days of MoM exposure (Figure 2). For detection of RV dysfunction at 1 year’s CA, a cutoff less than 27 days of MoM was found to have an AUC of 0.912 (95% CI, 0.851-0.973) (Figure 3). Compared with full-term infants at age 1 year, all measures of RV function and RV morphology were decreased in the preterm group, except RV major length (eTable 1 in the Supplement). There was no difference in measures of RV function and RV morphology at 32 weeks’ PMA (eTable 3 in the Supplement). At 36 weeks’ PMA, multivariate analysis found that for each additional week of MoM exposure there was enhanced RV function (eg, right longitudinal strain: β, 0.016; 95% CI, 0.002 to 0.031; *P* < .001) and increased RV morphology (eg, systolic area: β, 0.023;95% CI, 0.014 to 0.042; *P* < .001).

### Pulmonary Hemodynamics

At 1 year’s CA, multivariate analysis found that for each additional week of MoM exposure there was decreased RV afterload, as measured by PAATi (β, −0.024; 95% CI, −0.044 to −0.012; *P* < .001), and increased coupling of the RV to its afterload (TAPSE/PAATi: β, −0.019; 95% CI, −0.034 to −0.003; *P* < .001) (Table 2). Linear regression analysis found negative correlation between PAATi at 1 year’s CA and days of MoM exposure (*r* = –0.99; *P* < .001) and positive correlation between TAPSE/PAATi and days of MoM exposure (*r* = 0.72; *P* < .001) (eFigure 2 in the Supplement). For detection of elevated PAATi at 1 year’s CA, a cutoff of less than 30 days of MoM was found to have an AUC of 0.903 (95% CI, 0.821-0.975) (eFigure 3 in the Supplement). Compared with full-term infants at age 1 year, PAATi was decreased and TAPSE/PAATi was increased in the preterm group. (eTable 1 in the Supplement). There was no difference in measures of PAATi or TAPSE/PAATi at 32 weeks’ PMA based on MoM exposure (eTable 2 in the Supplement). At 36 weeks’ PMA, multivariate analysis found that for each additional week of MoM exposure there was decreased PAATi (β, –0.0021; 95% CI, –0.0034 to –0.0010; *P* < .001) and increased TAPSE/PAATi (β, –0.0017; 95% CI, –0.0046 to –0.0001; *P* < .001).

In post hoc analysis, we determined that to detect RV dysfunction, LV dysfunction, and elevated RV afterload, cutoff values greater than 27, 26, and 30 days of MoM, respectively, resulted in high sensitivity and specificity with large areas under the ROC curve (Figure 3 and eFigure 3 in the Supplement). As such, In subanalysis we dichotomized the cohort by high and low MoM exposure, defined as greater than 28 days of MoM (eFigure 1 in the Supplement) and compared the values between MoM exposure groups and to full-term infants at 1 year and 1 month of age. (eAppendix, eFigure 5, eFigure 6, and eFigure 7 in the Supplement). Finally, eTable 4 in the Supplement summarizes the comparisons between infants discharged on MoM vs infants discharged on formula.

### Donor Human Milk Exposure

In the model, there was no impact of exposure to DHM on cardiac performance at 1 year’s CA from the full cohort of preterm infants. To separate the association of DHM from MoM on the primary outcomes, we did a subanalysis of preterm infants who received DHM from the infants who received less than 28 days of MoM (see eTable 5 and the eAppendix in the Supplement), and compared cardiopulmonary measures to weeks of DHM exposure. At 1 year’s CA, multivariate analysis found that for each additional week of DHM exposure (in this group with low exposure to MoM), there were increased measures of RV function (eg, right longitudinal strain: β, 0.036; 95% CI, 0.012 to 0.071; *P* < .001) and morphology (eg, systolic area: β, 0.029; 95% CI, 0.011 to 0.055; *P* < .001), even after adjustments for gestational age at birth, sex, heart rate, and common comorbidities (BPD, late PH, and necrotizing enterocolitis) (eTable 5 in the Supplement). There was no difference in measures of LV function, LV morphology, RV afterload, or RV coupling at any time point over the first year of age based on DHM exposure.

## Discussion

To our knowledge, this study provides the first evidence of an association between early postnatal nutrition in preterm infants and cardiac performance over the first year of age. Preterm infants exposed to more MoM during the early neonatal period had greater LV and RV function and structure with lower pulmonary pressures and enhanced RV to PA coupling at age 1 year, with all measures approaching those seen in controls born full-term. There were no differences in any of the cardiac indices at 32 weeks’ PMA, but at 36 weeks’ PMA RV function was greater and pulmonary pressures lower with increased MoM exposure. This study’s findings suggest that the type of enteral nutrition may play a role in mitigating adverse cardiac programming during early life for preterm infants.

The cardiovascular system of preterm infants, particularly those born before 29 weeks’ gestation, is characterized by substantial myocardial systolic and diastolic dysfunction coupled with a maladaptive and immature vasculature driven by several unique developmental and maturational patterns.^7^ Long-term cardiovascular consequences following preterm birth, including heart failure, ischemic heart disease, poor exercise tolerance, hypertension, cardiometabolic disease, and persistent PVD,^1^ may be associated with the cardiovascular phenotypes that develop over the first year of age in individuals born preterm.^7^ A meta-analysis found that individuals born preterm have persistently smaller ventricular dimensions, lower LV diastolic function that worsens with age, RV systolic impairment across all developmental stages, and an accelerated rate of LV hypertrophy from childhood to adulthood.^24^ These collective observations have prompted a renewed interest in discerning potential interventions aimed at lessening or reversing those cardiovascular changes.

As the benefits of human breast milk have been linked to enhanced brain^25^ and lung development,^26^ investigators have explored early optimal nutrition consisting of mainly human milk during the neonatal period as a mitigating factor and an essential component of improved cardiovascular health.^8,9,27^ Follow-up data of premature neonates who were enrolled in a nutritional intervention trial in the United Kingdom in the 1980 offer the clearest association between early nutrition and long-term cardiovascular health. Preterm infants receiving exclusive MoM in the neonatal period had lower blood pressure during adolescence^27^ and a more favorable cardiac structural profile during young adulthood.^8^ Despite the emergence of this literature regarding enhanced long-term cardiovascular health, there is lack of robust data regarding the relationship of human breast milk and cardiovascular development over the first year of age in preterm infants.^9^ In our study, cardiovascular function and morphology at 32 weeks’ PMA were not associated with MoM exposure, but by age 1 year cardiovascular performance was enhanced in infants with higher MoM exposure. Interestingly, LVMi was increased and RWT decreased in infants with higher MoM exposure by age 1 year, suggesting a reversal of arrested cardiac growth, further supported by the large chamber volumes. Taken altogether, our study provides further evidence supporting the beneficial association of early MoM exposure with cardiovascular health in the preterm infant population.

The maturational patterns of cardiovascular performance over the first year of age are ventricular specific, with common neonatal morbidities (eg, BPD and PH) being associated with decreased RV function and pulmonary hemodynamics.^11,12^ Investigations in adults who were born preterm have shown that developmental changes appear to be more adverse in the RV than the LV^6^ with the degree of prematurity as the strongest risk factor for these changes.^11,28^ In our study, the magnitude of RV function was enhanced as early as 36 weeks’ PMA in infants with higher MoM exposure, whereas LV function was unaffected. It has been proposed that this so-called interdependence or coupling of the lungs and the RV is the reason preterm infants fed exclusive human milk had enhanced RV volumes and function as young adults.^8^ Similarly, in our study RV afterload was lower and coupling improved in infants with higher MoM exposure at 36 weeks’ PMA and persisted to 1 year’s CA, highlighting the potential mitigating association of early MoM with anticipated abnormal coupling in preterm infants.

Our understanding of the cardioprotective effects of human milk feeding in preterm infants is only at an early stage, and it supports the well-established developmental origins of health and disease framework that states early life environmental factors can have a modulating factor in chronic diseases in the future.^29^ The improved survival of extremely preterm infants has led to increased recognition of the importance of deciphering cardiovascular health during and beyond the neonatal period. This study adds to the already known benefits of human milk exposure in the high-risk preterm population^8,9^ and appears to be consistent with findings in full-term infants.^30^ One of the key missing pieces, however, is the potential mechanism(s) through which MoM may modulate long-term cardiac phenotype in the prematurely born infant. Those potential mechanisms are detailed at length elsewhere^9^ but may include growth factors and hormones (such as VEGF and adiponectin),^31^ immunological and inflammatory modulators (immunoglobulins, lactoferrin and pluripotent stem cells),^32^ and human milk oligosaccharides.^33^ Identifying the exact mechanism may pave the way for further specific therapeutic interventions targeting the cardiovascular response to prematurity.^9^

### Limitations

This study has some limitations. Despite the changes in cardiac performance based on MoM exposure, causality cannot be proven from this post hoc, nonrandomized design with the potential for unknown confounders limiting the interoperability of the data. First, there was a loss of follow-up from the original PROP study and risk of ascertainment bias due to the prospective nature of following this cohort from birth to 1 year’s CA.^8^ Second, although there were no differences in maternal and infant demographics characteristics or clinical databased on MoM exposure, the lack of association between the exposure and the common neonatal morbidities may be related to sample size. For example, we did not find a significant difference in NEC rates based on MoM exposure, despite its reported association. Third, we did not account for environmental or social factors that could impact the primary outcome. Specifically in this study, there were more White mothers who provided MoM compared with Black mothers. Although race comparisons were not significant, there could be inherent predictive elements among Black neonates with adverse cardiopulmonary outcomes related to allostatic load or other race factors.^34^ Future studies are needed to explore potential association of racial disparities in MoM exposure on cardiac development following premature delivery. Fourth, the nutritional intake of the full-term infants was not captured preventing matched odds ratio analysis. Finally, with the availability of DHM, exclusive formula feeding is no longer a common clinical practice in neonatal intensive care units in the developed world.^35^ DHM is pasteurized leading to a dramatic reduction in the bioactive properties of human milk, but they are not completely eradicated, potentially offering a similar explanation with MoM to enhanced cardiovascular performance.^36^ Future research is needed to investigate DHM and its mechanistic underpinnings as a modifiable intervention for improving cardiovascular performance over the first year.

## Conclusions

This study found a beneficial association between human milk consumption and cardiovascular health and early cardiovascular development in preterm infants. Preterm infants with higher consumption of mother’s own milk in the neonatal period had enhanced cardiac performance that was identified as early as 36 weeks’ PMA and became more established by age 1 year. Breast milk consumption in the early neonatal period may play a role in normalization of aspects of the preterm cardiac phenotype, and supports the use of human milk to diminish long-term cardiovascular disease risk in individuals who were born preterm.

## Notes

Supplement. **eAppendix. **Supplementary Materials **eTable 1. **Comparison of Cardiac Parameters at 1 Year of Age **eTable 2. **Comparison of Cardiac Parameters at 1 Month of Age **eTable 3. **Multivariate Analysis of Cardiac Parameters at 1 Month of Age **eTable 4**. Comparison of Cardiac Parameters at 1 Year of Age based on Discharged Nutrition **eTable 5. **Donor Human Milk and Cardiac Parameters at 1 Year Corrected Age **eFigure 1. **Histogram Distribution of Days of Mother’s Own Breast Milk **eFigure 2. **Linear Regression Analysis at 1 Year of Age for Pulmonary Hemodynamics **eFigure 3. **Receiver Operator Curves to Detect Elevated Pulmonary Vascular Resistance **eFigure 4. **Comparison of Cardiac Measures at 1 Year of Age Between Preterm and Term Infants **eFigure 5. **Comparison of Pulmonary Hemodynamics at 1 Year of Age Between Preterm and Term Infants **eFigure 6. **Maturational Patterns of Cardiac Mechanics **eFigure 7. **Maturational Patterns of Pulmonary Hemodynamics
eReferences
