# Supplementary material for: Cardiac Performance in the First Year of Age Among Preterm Infants Fed Maternal Breast Milk
Source: JAMA Netw Open. 2021 Aug 27;4(8):e2121206. doi: 10.1001/jamanetworkopen.2021.21206 (PMC8397926; doi:10.1001/jamanetworkopen.2021.21206)
Supplement: Supplement. — eAppendix. Supplementary Materials eTable 1. Comparison of Cardiac Parameters at 1 Year of Age eTable 2. Comparison of Cardiac Parameters at 1 Month of Age eTable 3. Multivariate Analysis of Cardiac Parameters at 1 Month of Age eTable 4. Comparison of Cardiac Parameters at 1 Year of Age based on Discharged Nutrition eTable 5. Donor Human Milk and Cardiac Parameters at 1 Year Corrected Age eFigure 1. Histogram Distribution of Days of Mother’s Own Breast Milk eFigure 2. Linear Regression Analysis at 1 Year of Age for Pulmonary Hemodynamics eFigure 3. Receiver Operator Curves to Detect Elevated Pulmonary Vascular Resistance eFigure 4. Comparison of Cardiac Measures at 1 Year of Age Between Preterm and Term Infants eFigure 5. Comparison of Pulmonary Hemodynamics at 1 Year of Age Between Preterm and Term Infants eFigure 6. Maturational Patterns of Cardiac Mechanics eFigure 7. Maturational Patterns of Pulmonary Hemodynamics eReferences [file jamanetwopen-e2121206-s001.pdf]

Supplemental Online Content

El-Khuffash A, Lewandowski AJ, Jain A, Hamvas A, Singh GK, Levy PT. Cardiac performance in the first year of age among preterm infants fed maternal breast milk. *JAMA Netw Open.* 2021;4(8):e2121206. doi:10.1001/jamanetworkopen.2021.21206

eAppendix. Supplementary Materials
eTable 1. Comparison of Cardiac Parameters at 1 Year of Age
eTable 2. Comparison of Cardiac Parameters at 1 Month of Age
eTable 3. Multivariate Analysis of Cardiac Parameters at 1 Month of Age
eTable 4. Comparison of Cardiac Parameters at 1 Year of Age based on Discharged Nutrition
eTable 5. Donor Human Milk and Cardiac Parameters at 1 Year Corrected Age
eFigure 1. Histogram Distribution of Days of Mother's Own Breast Milk
eFigure 2. Linear Regression Analysis at 1 Year of Age for Pulmonary Hemodynamics
eFigure 3. Receiver Operator Curves to Detect Elevated Pulmonary Vascular Resistance
eFigure 4. Comparison of Cardiac Measures at 1 Year of Age Between Preterm and Term Infants
eFigure 5. Comparison of Pulmonary Hemodynamics at 1 Year of Age Between Preterm and Term Infants
eFigure 6. Maturational Patterns of Cardiac Mechanics
eFigure 7. Maturational Patterns of Pulmonary Hemodynamics
eReferences

This supplemental material has been provided by the authors to give readers additional information about their work.

© 2021 El-Khuffash A et al. *JAMA Network Open.*

eAppendix. Supplementary Materials

EXPANDED METHODS

Study Participants and Timing of echocardiograms

The preterm-born and term-born cohorts were both prospectively recruited at birth and followed to 1 year of age. The preterm infants had echocardiograms performed at 32 weeks postmenstrual age (PMA), 36 weeks PMA and 1 year corrected age (CA). Term infants were assessed at 1 month and 1 year of age. The timings of the echocardiographic studies at 32 and 36 weeks PMA in the preterm infants were pre-selected to avoid the transitional period of cardiopulmonary and clinic uncertainty and early morbidity and associated mortality with extreme preterm birth.[1,2] Since the preterm cohort included infants born between 23 and 0/7 weeks and 28 and 6/7 weeks gestation at birth, we choose to study all infants at a common PMA and a common CA to optimize the determination of the impact of gestational and chronological age on cardiac performance at a specific developmental stage and allow for comparison of measures between term and preterm infants by post-gestational days from birth.[1,2] As previously described,[3] preterm infants received their first echocardiogram at a median of 38 days (interquartile range of 33 to 45 days). Term infants had the initial echocardiogram at a median of 32 days (interquartile range of 31 to 35 days). While, the PMA is different between the preterm-born infants (32 weeks PMA) and term-born infants (range between 41 and 44 weeks PMA) at the 1 month echocardiogram, comparing these time points by median days of age allowed for maturational comparisons over the first year of age.[4] Preterm infants had the second echocardiogram at 36 weeks PMA with a median of 67 days (interquartile range of 60 to 76 days). Preterm infants had the third echocardiogram (~ 1 year CA) at a median of 494 days (interquartile range of 452 to 521 days). The CA in days for all preterm infants was 381 days (355 to 424 days). The term infants received the 1-year of age echocardiogram at a median of 378 days (interquartile range of 365 to 399 days).

© 2021 El-Khuffash A et al. *JAMA Network Open.*

Exclusion criteria for the preterm-born and term-born populations have been previously described.[3,5,6] In brief, preterm infants were excluded if they had any suspected congenital anomalies of the airways, congenital heart disease, fetal growth restriction (FGR)[7] or small for gestational age (birth weight < 10th centile for gestation). The exclusion criteria for the control cohort were multiple gestation pregnancy, maternal smoking, maternal diabetes, antepartum hemorrhage, pre-eclampsia, or proven chorioamnionitis. Infants with FGR, perinatal acidosis (umbilical cord pH < 7.10), suspected congenital or chromosomal anomalies, neuromuscular disorders, abnormal cardiac rhythms, heart failure, and infants readmitted to the hospital within the first year of age were also excluded.

Defining High vs. Low Mothers Own Milk Exposure Groups

The PROP study design defined high breast milk exposure as greater than 28 days (4 weeks) of consumption of mother's own milk (MoM) feeds before 36 weeks PMA.[8] The 28-day cut-off mark was chosen to 1) avoid any concerns with missing data and 2) not bias against infants with feeding difficulties **(eFigure 1).** In post-hoc analysis, we determined that to detect right ventricular (RV) dysfunction, left ventricular (LV) dysfunction, and elevated RV afterload, cut-off values > 27, 26, and 30 days of MoM, respectively, resulted in high sensitivity and specificity with large areas under the ROC curve **(Figure 3 and eFigure 3)**. As such, in sub-analysis we dichotomized the cohort by high and low MoM exposure, defined as greater than 28 days of MoM and compared the values between MoM exposure groups and to term born infants at 1 year and 1 month of age (**eTable 1** and **eTable 2,** respectively). At the time of the original PROP study design in 2009, most of the data supported the effect of three months of breastfeeding in term infants, with little to no data on the threshold on impact for preterm infants. As such, preterm-based outcome papers have used breast milk at discharge in their analysis (which likely reflects a minimum of three months of the exposure at that point). Since the majority of extreme low birth weight preterm infants are discharged after 36 weeks post-menstrual age

(which is >3 months for an extreme preterm infant), we also analyzed the cohort based on their discharge nutritional status, MoM vs. bovine formula. To note, as per standard center protocol, infants born less than 29 weeks gestation received bovine fortifiers when they reach 100 milliliter per kilogram per day of feeds and remained on fortification through discharge.

Echocardiographic Measures

Echocardiograms were acquired with commercially available ultrasound imaging systems (Vivid 7 and E9; General Electric Medical Systems, Milwaukee, Wisconsin). One designated experienced pediatric cardiac sonographer obtained all the echocardiographic images using a phased array transducer (5–12 megahertz).[2] Echocardiographic measurements for LV and RV functional, morphometric parameters, and estimates of pulmonary hemodynamics were obtained in both term and preterm infants with each echocardiographic study. All measures were acquired and analyzed using previously published image acquisition and data analysis protocols from our laboratories.[2,9] All measures were generated according to guidelines of the American Society of Echocardiography[10,11] and recent validated protocols in neonates.[12] Echocardiographic data have previously been published from the preterm cohorts,[1,2,4,13,14] but the measurements had not been previously assessed in relationship to daily milk consumption. We have also previously demonstrated excellent reproducibility in term and preterm infants with each of the measures described in this section.[2,4,13-15]

LV Function

LV function was characterized by the quantification of two dimensional-speckle tracking derived deformation imaging.

A. *Deformation:* LV free wall longitudinal strain (%) and systolic early, and late diastolic strain rate (1/sec) were assessed with two-dimensional speckle tracking echocardiography using previously published image acquisition and data analysis protocols from our laboratories.[2,9] Longitudinal strain

© 2021 El-Khuffash A et al. *JAMA Network Open.*

is a measure of the maximal shortening of myocardial longitudinal length during systole compared to the resting length in diastole. The speed at which the myocardial wall deforms and then returns to baseline is measured as the time derivate of strain in systole and diastole, referred to as systolic and early and late diastolic strain rate, respectively. A frame rate to heart rate ratio between 0.7 and 0.9 frames/sec per beats per minute was utilized to optimize myocardial speckle tracking and mechanical event timing.[9] Peak strain for each index was measured as end-systolic strain at the closure of the aortic valve.[16] Two observers, who were blinded to the maternal and infant clinical and cardio-respiratory conditions, analyzed deformation using vendor customized commercially available software (EchoPAC; General Electric Medical Systems, Waukesha, WI, USA, version 112).

LV Morphology

LV morphology was assessed LV mass index (LVMi) and relative wall thickness (RWT) and according to previously published guidelines.[11] The estimation of LVMi and RWT were derived from LV measurements obtained by two-dimensional guided M-mode echocardiography using the parasternal short-axis view at the level of the papillary muscles. End diastole was defined as the time of maximum LV dimension. We measured end diastole interventricular septal thickness (IVSd), left ventricular (LV) posterior wall thickness (LVPWd), and LV dimension at end diastole (LVEDD) and end systole (LVESD) over three consecutive cardiac cycles and averaged the values.

A. LVM was estimated by the Devereux equation, LVM (grams) = $0.8\{1.04\,[(LVEDD + LVPWd + IVSd)^{3} - (LVEDD)^{3}]\} + 0.6$.[17] We indexed LVM to allow comparisons between term and preterm infants with different body sizes. In this study, LVM was normalized to height to the allometric power of 2.7 to obtain LVMi.[18] Although whether to use height, weight, or BSA as the indexing term is unclear in children, it has been previously shown that LV mass centile curves indexed to weight, length, and BSA are all practical method to assess LV morphology in preterm infants.

© 2021 El-Khuffash A et al. *JAMA Network Open.*

Accordingly, we included these analyses to assess LV morophology.[19] We used the Haycock formula for calculation of body surface area: weight$^{0.5378}$ × height$^{0.3964}$ × 0.024265.[20] Since altered LV geometry in preterm infants is also associated with steroid use,[15] we also accounted for its postnatal use in the model.

B. Relative wall-thickness (RWT) was calculated using two formulas: (1) RWT equals twice the posterior wall thickness (LVPWd) over LVEDD; (2) RWT equals the ratio of the sum of LVPWd and IVSd over LVEDD.[21] In the result section, we present RWT determined with twice the LVPWd over LVEDD rather than RWT equals the ratio of the sum of LVPWd plus IVSd due to the potential interference by tricuspid valve tissue and right ventricular trabeculations with septal measurement.[15]

RV Function

The major contribution to ejection fraction and stroke volume for the RV during systole is provided by the dominant deep longitudinal fiber layer, which makes up 80% of the RV free wall thickness. In this study, RV performance was assessed using measures that assess changes in RV longitudinal shortening from one of three distinct approaches in neonates:[12] (i) change in cavity dimensions (e.g. fractional are of change, FAC) (ii) displacement and velocity of single point along the myocardial wall (e.g. tricuspid annular plane systolic excursion, TAPSE); and (iii) deformation of a segment of the wall (strain and strain rate imaging).[22]

A. FAC (%) reflects a change in cavity size and is a surrogate of RV ejection fraction.[1] FAC is calculated by tracing the endocardial borders, including the trabeculations with the area, at end-diastole and end-systole and applying these measurements to the following equation: [(end-diastole area - end-systolic area)/ end-diastole area] x 100. FAC was measured from the apical 4 –chamber view.

B. TAPSE (mm) measures the longitudinal distance (displacement) that the tricuspid valve annulus moves during systole. Contraction of the deep longitudinal fibers of the RV free wall pull the

© 2021 El-Khuffash A et al. *JAMA Network Open.*

tricuspid valve from the base to the apex during systole. TAPSE measures this dominant component of RV systolic performance and provides an estimate of longitudinal myocardial shortening. For this study, TAPSE was obtained at the level of the lateral TV annulus using M-Mode.

C. The same deformation approach utilized from the LV was applied to the RV, but assessment of RV longitudinal strain and strain rate images were acquired from an RV focused apical four chamber view according to previous published protocols.[2,13]

RV Morphology

RV morphology was assessed with RV areas[1,23] and RV linear dimensions.[3]

A. RV areas were assessed from the RV focused apical 4-chambier view in systole and diastole.

B. RV linear dimensions were measured at the basal and mid ventricular levels and in the longitudinal direction from the apical 4-chamber view at end-diastole.[4,10,11]

RV Afterload/Pulmonary Hemodynamics

We measured several different markers of RV afterload to account for its two main components, pulmonary vascular resistance and compliance.[24] Specifically, we measured pulmonary artery pressure derived by both the tricuspid regurgitation jet velocity, when available, and RV systolic time intervals.

A. Pulmonary artery systolic pressure was estimated by the tricuspid regurgitation jet velocity, when available

B. Pulmonary artery systolic pressure was estimated by RV systolic time intervals, also known as pulmonary artery acceleration time (PAAT) and its ratio to right ventricle ejection time (RVET) to PAAT (PAATi).[14] PAAT and PAATi have been validated against cardiac catheterization in children and neonates,[24] and maturational patterns have recently been established in healthy children[25] and preterm infants.[14] Specific methods for acquisition are described elsewhere.[14]

© 2021 El-Khuffash A et al. *JAMA Network Open.*

RV Function to RV Afterload Coupling

The RV and pulmonary arterial (PA) circulation function as one unit, commonly referred to as the RV-PA axis.[26] Recent evidence suggests an index of ventriculo-arterial coupling can serve as a comprehensive measure of RV- PA axis with different neonatal outcomes.[27] Accordingly, we assessed the relationship of TAPSE (a measure of RV longitudinal shortening) to PAATi (a comprehensive measure of RV afterload) as a surrogate of RV-PA coupling.[28,29]

Common Neonatal Morbidities

The following additional common neonatal outcomes were evaluated: intraventricular hemorrhage (classified according to Papile Classification),[30] retinopathy of prematurity (stage 2 or higher), necrotizing enterocolitis (Bells Stage II, with radiological evidence of pneumatosis), BPD (we used a modified version of 2001 National Institutes of Health workshop definition of bronchopulmonary dysplasia),[6] and late PH. Preterm infants were classified with late pulmonary hypertension if they had two or more of the following conventional echocardiographic measures of pulmonary hemodynamics at any of the echocardiograms: 1) an estimated right ventricle systolic pressure > 40 mmHg, using the Doppler velocity of tricuspid regurgitation, 2) a ratio of right ventricle systolic pressure to systemic blood pressure > ½; 3) bidirectional or right to left shunt at the atrial level or patent ductus arteriosus**;** 4) septal wall flattening as defined as decreased septal curvature into the RV at end systole, or 5) dilated/hypertrophy of the right ventricle and right atrial morphology.[4,14]

Sample Size Justification: Power Analysis

To overcome the limitations of enrolling enough patients at specific gestational ages with certain weights to detect statistically significant differences, our initial calculations on the basis of preliminary analysis and literature searches determined that we needed 150 patients to detect a 20% difference in LV

© 2021 El-Khuffash A et al. *JAMA Network Open.*

systolic and RV systolic strain to expect moderate associations between MoM and strain (r = 0.3). Our design maintains 90% power for this effect size with n = 150 and 95% power with n = 170. The larger sample size allows power to detect variability in rates of change in our trajectory models, as well as 90% power for group differences of d = 0.43 and r = 0.20. The sample size of 170 assumes a missing rate of 10% (5% because of late deaths and 5% because of loss to follow-up) that would allow for a power of >80% to detect a significant association at an odds ratio of ≥1.30 for each 1-SD increase in RV and LV strain associated with gestational age. This calculation assumed no measurement errors and no correlation between strain and other covariates and a conservative 50% rate of BPD and 20% PH. In addition to account for the potential limitation of enrolling enough patients at specific gestational ages with certain weights to detect statistically significant differences, we decided to also generate maturational patterns and compare them between preterm-born infants in the high MoM, preterm-born infants in the low MoM, and term-born control infants at one year of age.

EXPANDED RESULTS

Characteristics of Cohorts

One hundred and eighty infants (80 born preterm and 100 born at term) were included in this study **(Figure 1).** In the original Prematurity and Respiratory Outcomes Program (PROP) at the Washington University School of Medicine site, 137 infants were recruited at birth. Of the 137 preterm infants recruited at birth, 13 died, two withdrew, and five transferred before completion of the study. At 32 and 36 weeks PMA, 117 infants were evaluated with an echocardiogram. Of the 117 alive at discharge, 37 were lost to follow-up and 80 preterm infants returned at 1 year CA. Only infants with echocardiograms at 32 weeks PMA, 36 weeks PMA and 1 year CA were included in this study. A cohort of 100 healthy, age- and sex-matched infants born at term were recruited for the control group and had echocardiograms performed at 1 month and 1 year of age. The median gestational age at birth of the 100 term-born infants

© 2021 El-Khuffash A et al. *JAMA Network Open.*

was 39.0 weeks (IQR 38.0 – 40.0) and the median birth weight was 3165 g (IQR 2900–3760). Of the 100 term infants, 54 were white and 46 were black.

Clinical Data Reporting

The clinical information that was not described in **Table 1** is detailed below in this supplement explanation. In this study, none of the preterm infants received inotropes, inhaled nitric oxide or other pulmonary vasodilators at any time point in the first year of age. Additionally, none of the preterm born infants were on respiratory support at 1 year CA. In our neonatal intensive care unit, all preterm infants born less than 29 weeks gestation received caffeine from birth through 34 weeks PMA, and none received it beyond 34 weeks PMA. Twenty-six preterm infants (33%) received postnatal steroids at least one time during their hospital course, of which 23 (88%) received them to treat lung disease beyond the first month of age. There was no significant difference in postnatal steroid administration based on MoM exposure (p=.13). Of the 23 infants that received postnatal steroids to treat lung disease beyond the first month of age, all were diagnosed with BPD. Of this subset, there was also no significant difference in postnatal steroid administration base on MoM exposure(p=.45). The three (12%) infants who did not develop BPD received steroids in the first month of age to facilitate a trial of extubation. At least one dose of diuretics was administered to 15 (18%) of the preterm infants during their neonatal course. All 15 were on a diuretic at 32 weeks PMA, but off by 36 weeks PMA and remained off at 1 year CA. There was no statistical difference in any of the cardiac indices between those infants that did and did not receive diuretics (p=.43), or postnatal steroids (p=.49), although the study was not adequately powered to answer these questions.

A patent ductus arteriosus (PDA) was present on echocardiogram at 32 weeks PMA in 20% (n=16) of infants. A PDA was present on echocardiogram at 36 weeks PMA in 10% (n=8) of group. At 1 year of age, a PDA was not present in any of the infants. There were no differences in cardiac function or morphology based on MoM exposure and presence of a PDA, adjusted for gestational age, sex, PDA size, diagnosis of BPD and late PH (p > .05 for all measures at 32 and 36 weeks PMA, and 1 year CA).

© 2021 El-Khuffash A et al. *JAMA Network Open.*

Similarly, there was no difference in measures of cardiac performance at 32 and 36 weeks PMA or 1 year CA based on MoM exposure and those that required medical (22/80, 28%) and/or surgical intervention (12/80, 15%) to augment closure of the PDA. None of the infants had their PDA closed with a transcatheter device as this study was performed prior to the availability of the transcatheter device at our center. There was no PDA present at 1 month or 1 year of age in the term population and no PDA present at 1 year of age in the preterm or term populations.

LV Morphology, RWT

At 1 year CA multivariate analysis demonstrated that for each additional week of MoM exposure there was decreased RWT (p<.001) **(Table 2)**. Linear regression analysis demonstrated negative correlation between RWT at 1 year CA and days of MoM exposure **(**r= -.62, p< .001). Compared to term infants at 1 year of age, RWT was larger in the preterm infants with high MoM exposure (p<.001). There was no difference in RWT at 32 weeks PMA or 36 weeks PMA base on the exposure of MoM **(eTable 3).**

Dose Response: High vs. Low MoM Cut-off

Of 80 preterm-born infants, 42 (53%) were exposed to > 28 days of MoM (high MoM) and 38 (47%) were exposed to < 28 days of MoM (low MoM). **eFigure 4** and **eFigure 5** dichotomize the group by high vs. low MoM exposure at 1 year CA and allow for comparison of the primary cardiopulmonary outcomes to term infants.

Maturational Patterns of LV performance

LV function, as characterized by the magnitude of LV longitudinal strain, increased from 32 weeks to 1 year of age in both the preterm and term infants. **(eFigure 6).** Similarly, LVMI also increased from 32 weeks to 1 year of age in both the preterm and term infants. Compared to term-born infants, all measures

of LV function were similar (p>.05) and LV morphology different (p<.05) at 32 weeks PMA with both exposure groups **(eTable 2).**

Maturational Patterns of RV Performance

The magnitudes of RV longitudinal strain, FAC, and TAPSE increased from 32 weeks PMA to 1 year CA in both the preterm and term infants **(eFigure 6).** RV morphology measures of systolic and diastolic areas, basal, mid-cavity, and apex-to-base linear dimensions also increased in both the preterm and term infants from 32 weeks postmenstrual age to 1 year CA (p<.01 for all measures). Compared to term infants, all preterm infants at 32 weeks PMA had decreased magnitudes of RV function and morphometric measures (P<.05 for all measures) **(eTable 2).**

Maturational Patterns of RV Afterload and Coupling

PAATi decreased from 32 weeks PMA to 1 year of CA in both the preterm and term infants (P<.05) **(eFigure 7)**. RV to PA coupling (TAPSE/PAATi) increased from 32 weeks PMA to 1 year CA in in both the preterm and term infants (P<.05) **(eFigure 7).**

Discharge Nutritional Status

eTable 4 summarizes the comparisons between infants discharged on MoM vs. infants discharged on Formula. There were 26 (33%) of infants' discharge on only MoM with a mean (standard deviation) of 59 days (10) on MoM. There were 54 (67%) of infants discharged on formula with a mean (standard deviation) of 22 days (15) on MoM. The mean and standard deviation in length of stay was similar between the two groups [91(23) vs. 94(22), p =.34]. Infants discharged on MoM had enhanced LV function, RV function, changes in LV and RV morphology, and measures of RV afterload at 1 year CA compared to infants discharged only on formula.

© 2021 El-Khuffash A et al. *JAMA Network Open.*

EXPANDED DISCUSSION

Additional Limitations

We did not explore factors that may influence a mother's ability to provide breast milk to her preterm infant during the neonatal period or at discharge.[31] Although preterm infants who received MoM at discharge in this study had enhanced cardiopulmonary function compared to infants who received formula at discharge, future work is needed to explore factors affecting breastfeeding outcomes in preterm infants and why a preterm infant would have higher exposure to MoM, as these factors themselves may also be linked to outcome measures.

© 2021 El-Khuffash A et al. *JAMA Network Open.*

eTable 1. Comparison of Cardiac Parameters at 1 Year of Age

	Preterm-born infant			Term-born		
	High MoM (n-=42)	Low MoM (n=38)	*P* value [a]	Controls (n=100)	*P* value [b]	*P* value [c]
Age at echocardiogram (days)	479 (461, 515) [d]	501 (469, 536) [d]	.17	378 (365, 399)	.35	.25
Weight at echocardiogram (kg)	9.7 (8.7, 11.1)	9.8 (9.0, 10.7)	.43	10.1 (9.1, 11.5)	.22	.43
Body surface area (m^2) [e]	.46 (0.42, 0.47)	.45 (0.43, 0.48)	.23	.45 (0.43, 0.49)	.46	.33
Heart rate (beats/minutes)	128 (115, 139)	127 (116, 137)	.36	125 (119-133)	.66	.26
Left ventricle function						
Longitudinal strain (%)	-20.2 (3.1)	-17.9 (2.4)	.02	-22.3 (2.4)	.006	.002
Longitudinal systolic strain rate (1/sec)	-1.6 (.23)	-1.3 (.4)	.04	-1.8 (.19)	<.001	<.001
Longitudinal early diastolic strain rate (1/sec)	2.5 (.34)	2.2 (.41)	.01	2.8 (.29)	<.004	<.01
Longitudinal late diastolic strain rate (1/sec)	2.3 (.31)	2.0 (.26)	.03	2.6 (.15)	<.002	<.01
Left ventricle Morphology						
LV mass index ($g/h^{2.7}$)	45 (4)	43 (3)	<.001	49 (5)	<.001	<.001
Relative wall thickness [f]	.31 (.07)	.33 (.04)	<.001	.28 (.04)	<.001	<.001
Right ventricle function						
Fractional area of change (%)	38 (4)	33 (3)	.01	43 (4)	<.001	<.001
Tricuspid annular plane systolic excursion (mm)	17 (3)	15 (4)	.03	19 (5)	.003	.005
Longitudinal strain (%)	-30.3 (2.4)	-27.4 (3.1)	.002	-33.6 (2.7)	<.001	<.001
Longitudinal systolic strain rate (1/sec)	-2.2 (.13)	-1.9 (.28)	.001	-2.8 (.31)	<.001	<.001
Longitudinal early diastolic strain rate (1/sec)	3.1 (.41)	2.6 (.27)	.004	3.5 (.34)	<.001	<.001
Longitudinal late diastolic strain rate (1/sec)	2.9 (.39)	2.3 (.25)	.007	3.6 (.28)	<.001	<.001
Right ventricle Morphology						
Basal length (cm)	2.4 (.49)	2.6 (.23)	.02	2.2 (.20)	<.001	<.001
Mid-cavity length (cm)	1.8 (.31)	2.0 (.22)	.03	1.6 (.24)	<.001	<.001
Major length (cm)	4.0 (.53)	4.0 (.34)	.34	4.1 (.39)	.28	.26
Systolic area (cm^2)	4.3 (.29)	3.6 (.31)	.01	4.5 (.21)	<.001	<.001
Diastolic area (cm^2)	6.5 (.23)	6.2 (.17)	.02	6.8 (.46)	<.001	<.001
Right ventricle afterload						
Pulmonary artery acceleration time (PAAT, ms)	79 (15)	63 (12)	<.001	97 (13)	<.001	<.001
PAATi (RVET / PAAT)	2.9 (.31)	3.8 (.42)	<.001	2.3 (.36)	<.001	<.001

Data presented at median (Interquartile range) or mean (standard deviation)

Abbreviations: LV, left ventricle; MoM, mother's own breast milk; PAATi, indexed pulmonary artery acceleration time; RV, right ventricle; RVET, right ventricle ejection time

[a] Preterm born high MoM vs. Preterm born low MoM. Comparisons adjusted for age, sex, bronchopulmonary dysplasia and late pulmonary hypertension.

[b] Term born vs. Preterm born high MoM. Comparisons adjusted for age, sex, bronchopulmonary dysplasia, late pulmonary hypertension, and necrotizing enterocolitis.

[c] Term born vs. Preterm born low MoM. Comparisons adjusted for age, sex, bronchopulmonary dysplasia, late pulmonary hypertension, and necrotizing enterocolitis.

[d] These numbers reflect actual days of age. Corrected age (days) for all preterm infants was 381 (355,424), for preterm infants in the high MoM was 388 (361, 419) and preterm infants in the low MoM was 387 (367, 428).

[e] We used the Haycock formula for calculation of body surface area: $weight^{0.5378} \times height^{0.3964} \times 0.024265$.

[f] Relative wall thickness (RWT) is presented as RWT = [2 x posterior wall thickness] / LV end diastolic dimensions.

© 2021 El-Khuffash A et al. *JAMA Network Open.*

eTable 2. Comparison of Cardiac Parameters at 1 Month of Age

	Preterm-born infant			Term-born		
	High MoM (n-=42)	Low MoM (n=38)	*P* value [a]	Controls (n=100)	*P* value [b]	*P* value [c]
Age at echocardiogram (days)	38 (35, 48) [d]	37 (31, 42) [d]	.14	32 (31, 35)	.45	.45
Weight at echocardiogram (kg)	1.4 (1.2, 1.6)	1.4 (1.2, 1.6)	.44	4.6 (4.3, 4.9)	.001	.001
Body surface area (m^2) [e]	.26 (0.24, 0.29)	.26 (0.23, 0.28)	.53	.27 (0.25, 0.28)	.23	.13
Heart rate (beats/minutes)	157 (145, 166)	158 (147, 170)	.46	155 (129, 166)	.26	.16
Left ventricle function						
Longitudinal strain (%)	-18.1 (3.2)	-18.3 (2.9)	.23	-19.1 (3.3)	.42	.56
Longitudinal systolic strain rate (1/sec)	-1.5 (.32)	-1.4 (.39)	.16	-1.6 (.22)	.47	.35
Longitudinal early diastolic strain rate (1/sec)	2.3 (.18)	2.4 (.36)	.43	2.5 (.32)	.49	.34
Longitudinal late diastolic strain rate (1/sec)	1.9 (.29)	1.9 (.35)	.35	2.0 (.31)	.39	.89
Left ventricle Morphology						
LV mass index ($g/h^{2.7}$)	38 (4)	39 (4)	.11	44 (5)	.001	.002
Relative wall thickness [f]	.33 (.05)	.34 (.06)	.64	.31 (.03))	.004	.004
Right ventricle function						
Fractional area of change (%)	32 (3)	32 (4)	.25	36 (3)	.005	.006
Tricuspid annular plane systolic excursion (mm)	8 (2)	8 (1)	.44	10 (2)	.03	.02
Longitudinal strain (%)	-18.2 (2.3)	-19.1 (3.1)	.22	-26.3 (2.5)	<.001	<.001
Longitudinal systolic strain rate (1/sec)	-1.8 (.31)	-1.8 (.29)	.35	-2.7 (.41)	.003	.003
Longitudinal early diastolic strain rate (1/sec)	2.1 (.43)	2.1 (.37)	.32	2.4 (.37)	.01	.02
Longitudinal late diastolic strain rate (1/sec)	1.6 (.22)	1.5 (.28)	.26	1.8 (.31)	<.001	<.001
Right ventricle Morphology						
Basal length (cm)	1.3 (.33)	1.3 (.31)	.22	1.7 (.23)	.001	.001
Mid-cavity length (cm)	1.1 (.23)	1.1 (.28)	.28	1.3 (.17)	.02	.03
Major length (cm)	2.1 (.37)	2.3 (.42)	.60	3.5 (.43)	.008	.008
Systolic area (cm^2)	1.7 (.31)	1.7 (.32)	.28	2.5 (.56)	.007	.006
Diastolic area (cm^2)	2.6 (.29)	2.6 (.34)	.40	3.8 (.41)	.006	.005
Right ventricle afterload						
Pulmonary artery acceleration time (PAAT, ms)	49 (12)	43 (15)	.35	75 (13)	<.001	<.001
PAATi (RVET / PAAT)	3.9 (.33)	4.4 (.54)	.22	2.9 (.31)	<.001	<.001

Data presented at median (Interquartile range) or mean (standard deviation)
Abbreviations: LV, left ventricle; MoM, mother's own breast milk; PAATi, indexed pulmonary artery acceleration time; RV, right ventricle; RVET, right ventricle ejection time

[a] Preterm born high MoM vs. Preterm born low MoM. Comparisons adjusted for age, sex, bronchopulmonary dysplasia and late pulmonary hypertension.

[b] Term born vs. Preterm born high MoM. Comparisons adjusted for age, sex, bronchopulmonary dysplasia, late pulmonary hypertension, and necrotizing enterocolitis.

[c] Term born vs. Preterm born low MoM. Comparisons adjusted for age, sex, bronchopulmonary dysplasia, late pulmonary hypertension, and necrotizing enterocolitis.

[d] The preterm cohort included infants born between 23 and 0/7 weeks and 28 and 6/7 weeks gestation at birth and we choose to study all infants at a common postmenstrual age (PMA) to optimize the determination of the impact of gestational and chronological age on cardiac performance at a specific developmental stage and allow for comparison of measures between term and preterm infants by post-gestational days from birth. The first echocardiogram in the preterm-born infants was acquired at 32 weeks PMA, a median of 38 days (interquartile range of 33 to 45 days).

[e] We used the Haycock formula for calculation of body surface area: $weight^{0.5378} \times height^{0.3964} \times 0.024265$.

[f] Relative wall thickness (RWT) is presented as RWT = [2 x posterior wall thickness] / LV end diastolic dimensions.

© 2021 El-Khuffash A et al. *JAMA Network Open.*

eTable 3. Multivariate Analysis of Cardiac Parameters at 1 Month of Age

Cardiac variable	B [a]	95% CI	P value
Left ventricle function			
Longitudinal strain (%)	.076	.038, .102	.33
Longitudinal systolic strain rate (1/sec)	.082	.048, .121	.23
Longitudinal early diastolic strain rate (1/sec)	.061	.028, .091	.36
Longitudinal late diastolic strain rate (1/sec)	.066	.042, .087	.51
Left ventricle Morphology			
LV mass index (g/h$^{2.7}$)	.121	.085, .145	.22
Relative wall thickness [c]	-.124	-.211, -.104	.42
Right ventricle function			
Fractional area of change (%)	.136	.122, .176	.27
Tricuspid annular plane systolic excursion (mm)	.132	.121, .146	.41
Longitudinal strain (%)	.111	.096, .132	.23
Longitudinal systolic strain rate (1/sec)	.093	.062, .115	.26
Longitudinal early diastolic strain rate (1/sec)	.105	.088, .143	.45
Longitudinal late diastolic strain rate (1/sec)	.103	.085, .141	.16
Right ventricle Morphology			
Basal length (cm)	-.199	-.331, -.103	.31
Mid-cavity length (cm)	-.123	-.212, -.071	.46
Major length (cm)	.079	-0.203, 0.199	.59
Systolic area (cm^2)	.031	.009, .062	.48
Diastolic area (cm^2)	.025	.011, .052	.47
Right ventricle afterload			
Pulmonary artery acceleration time (PAAT, ms)	.031	.009, .051	.38
PAATi (RVET / PAAT)	-.023	-.034, -.019	.12

[a] Multivariate analyses reported are adjusted absolute differences in means that correspond to a one week increase in mother's own breast milk (MoM) exposure. Comparisons adjusted for age, sex, bronchopulmonary dysplasia, late pulmonary hypertension, and necrotizing enterocolitis.
LV, left ventricle; PAATi, indexed pulmonary artery acceleration time; RV, right ventricle; RVET, right ventricle ejection time.
[b] Relative wall thickness (RWT) is presented as RWT = [2 x posterior wall thickness] / LV end diastolic dimensions.

© 2021 El-Khuffash A et al. *JAMA Network Open.*

eTable 4. Comparison of Cardiac Parameters at 1 Year of Age based on Discharged Nutrition

	MoM (n=26)	Formula (n=54)	*P* value
Left ventricle function			
Longitudinal strain (%)	-21.4 (2.5)	-18 (1.9)	.01
Longitudinal systolic strain rate (1/sec)	-1.7 (.42)	-1.4 (.33)	.02
Longitudinal early diastolic strain rate (1/sec)	2.6 (.38)	2.2 (.31)	.01
Longitudinal late diastolic strain rate (1/sec)	2.3 (.29)	1.9 (.18)	.02
Left ventricle Morphology			
LV mass index ($g/h^{2.7}$)	45 (4)	42 (3)	.006
Relative wall thickness [a]	0.31 (.03)	0.33 (.05)	.009
Right ventricle function			
Fractional area of change (%)	39 (5)	33 (4)	.003
Tricuspid annular plane systolic excursion (mm)	17 (3)	15 (2)	.01
Longitudinal strain (%)	-32.4 (3.2)	-27.3 (3.3)	.002
Longitudinal systolic strain rate (1/sec)	-2.4 (.21)	-2.0 (.35)	.008
Longitudinal early diastolic strain rate (1/sec)	3.1 (.32)	2.8 (.26)	.004
Longitudinal late diastolic strain rate (1/sec)	2.9 (.44)	2.4 (.32)	.003
Right ventricle Morphology			
Basal length (cm)	2.4 (.38)	2.5 (.18)	.02
Mid-cavity length (cm)	1.7 (.37)	1.9 (.23)	.01
Major length (cm)	4.1 (.34)	4.0 (.23)	.38
Systolic area (cm^2)	4.4 (.41)	3.7 (.27)	.01
Diastolic area (cm^2)	6.7 (.31)	6.3 (.26)	.02
Right ventricle afterload			
Pulmonary artery acceleration time (PAAT, ms)	80 (14)	65 (11)	.001
PAATi (RVET / PAAT)	2.9 (.35)	3.5 (.29)	.002

LV, left ventricle; MoM, mother's own breast milk; PAATi, indexed pulmonary artery acceleration time; RV, right ventricle; RVET, right ventricle ejection time.
[a] Relative wall thickness (RWT) is presented as RWT = [2 x posterior wall thickness] / LV end diastolic dimensions.

© 2021 El-Khuffash A et al. *JAMA Network Open.*

eTable 5. Donor Human Milk Multivariate Analysis of Cardiac Parameters at 1 Year Corrected Age

Cardiac variable	B [a]	95% CI	P value
Left ventricle function			
Longitudinal strain (%)	.036	.016, .093	.26
Longitudinal systolic strain rate (1/sec)	.041	.027, .103	.44
Longitudinal early diastolic strain rate (1/sec)	.052	.019, .082	.51
Longitudinal late diastolic strain rate (1/sec)	.039	.021, .063	.42
Left ventricle Morphology			
LV mass index (g/h$^{2.7}$)	.061	.043, .093	.17
Relative wall thickness [b]	-.091	-.132, -.062	.32
Right ventricle function			
Fractional area of change (%)	.084	.061, .123	<.001
Tricuspid annular plane systolic excursion (mm)	.091	.073, .121	<.001
Longitudinal strain (%)	.036	.012, .071	<.001
Longitudinal systolic strain rate (1/sec)	.061	.033, .097	<.001
Longitudinal early diastolic strain rate (1/sec)	.075	.043, .122	<.001
Longitudinal late diastolic strain rate (1/sec)	.056	.028, .089	<.001
Right ventricle Morphology			
Basal length (cm)	-.059	-.091, -.021	<.001
Mid-cavity length (cm)	-.023	-.089, -.005	<.001
Major length (cm)	.059	-0.03, 0.93	.32
Systolic area (cm^2)	.029	.011, .052	<.001
Diastolic area (cm^2)	.031	.008, .049	<.001
Right ventricle afterload			
Pulmonary artery acceleration time (PAAT, ms)	.042	.019, .072	.34
PAATi (RVET / PAAT)	-.034	-.052, -.009	.39

[a] Multivariate analyses reported are adjusted absolute differences in mean that correspond to a one week increase in donor human milk exposure. Comparisons adjusted for age, sex, bronchopulmonary dysplasia, late pulmonary hypertension, and necrotizing enterocolitis.
LV, left ventricle; PAATi, indexed pulmonary artery acceleration time; RV, right ventricle; RVET, right ventricle ejection time.
[b] Relative wall thickness (RWT) is presented as RWT = [2 x posterior wall thickness] / LV end diastolic dimensions.

© 2021 El-Khuffash A et al. *JAMA Network Open.*

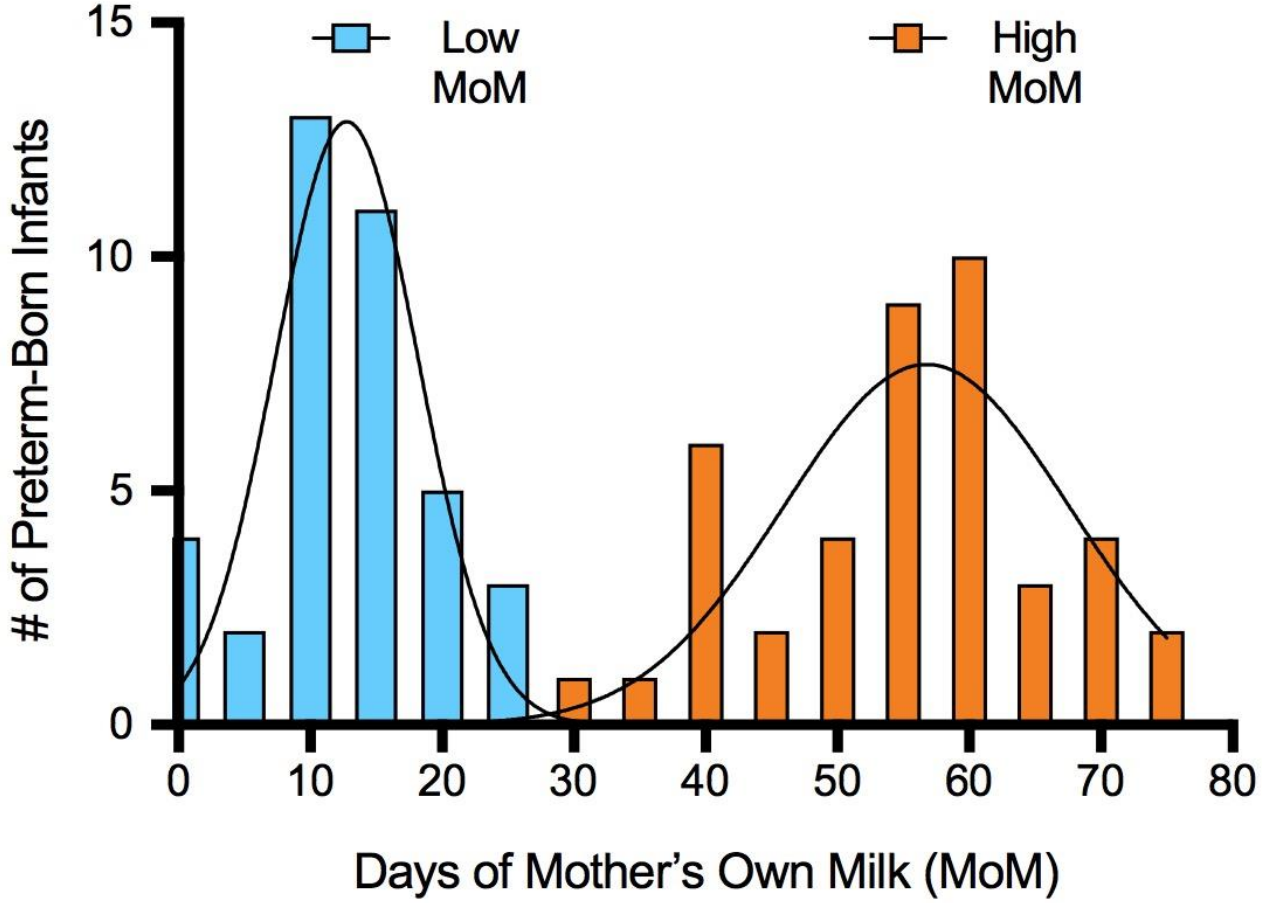

eFigure 1. Histogram of distribution of days of mother's own milk (MoM). The low MoM exposure (n=38) is in orange. The high MoM exposure group (n=42) is in blue.

© 2021 El-Khuffash A et al. *JAMA Network Open.*

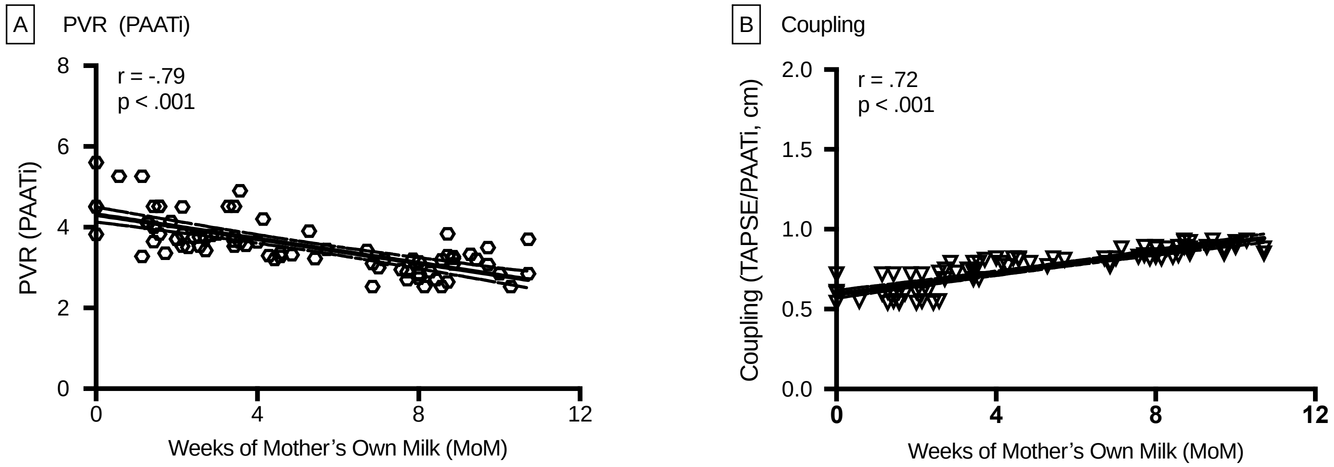

eFigure 2. Linear Regression Analysis at 1 Year of Age for A) PVR and B) Coupling. PVR, pulmonary vascular resistance. PAATi, indexed pulmonary artery acceleration time defined as the ratio of right ventricle ejection time (RVET) to PAAT. TAPSE, tricuspid annular plane systolic excursion.

© 2021 El-Khuffash A et al. *JAMA Network Open.*

Pulmonary Vascular Resistance (PAATi)

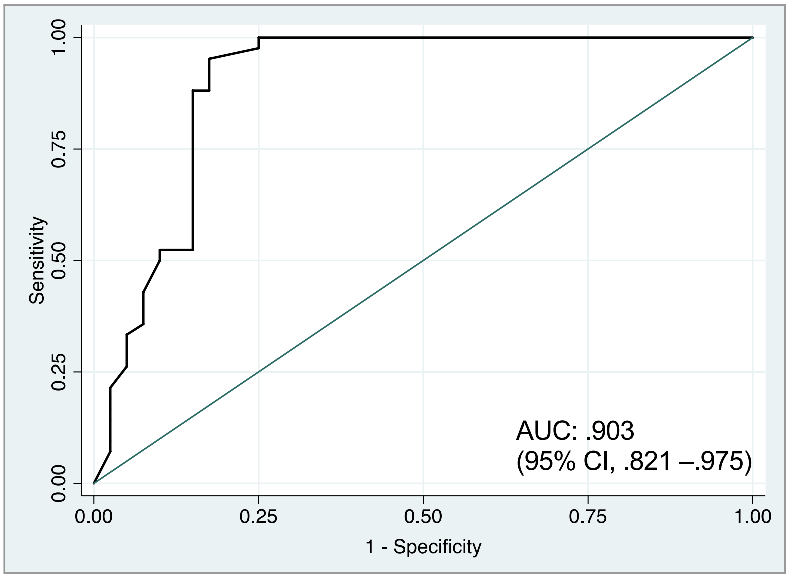

eFigure 3. Receiver operator curve analysis to determine specific cutoff values of days of mother's own milk in predicting elevated pulmonary vascular resistance. For detection of elevated pulmonary vascular resistance (indexed pulmonary artery acceleation time, PAATi > 3) at 1 year CA, a cut-off < 30 days of mother's own milk exposure resulted in sensitivity of 85% and specificity of 85% with an AUC of .903 (95% CI, .821 –.975)

© 2021 El-Khuffash A et al. *JAMA Network Open.*

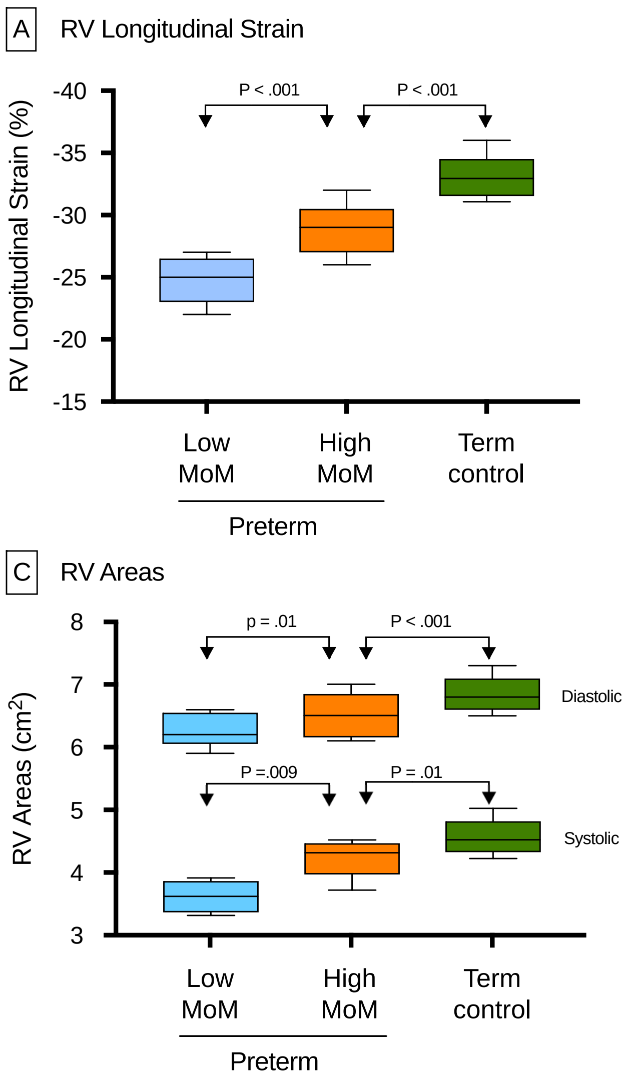

eFigure 4. Preterm-born infants in the high mother's own milk (MoM) exposure group (orange) had increased right and left ventricular strain (A and B), and right ventricular areas (C), and left ventricular mass index, LMVI, g/h$^{2.7}$, (D) compared with preterm-born infants in the low MoM exposure group (blue) at 1 year corrected age. Term-born control infants are shown in green at 1 year of age. High MoM is defined as > 28 days of MoM exposure

© 2021 El-Khuffash A et al. *JAMA Network Open.*

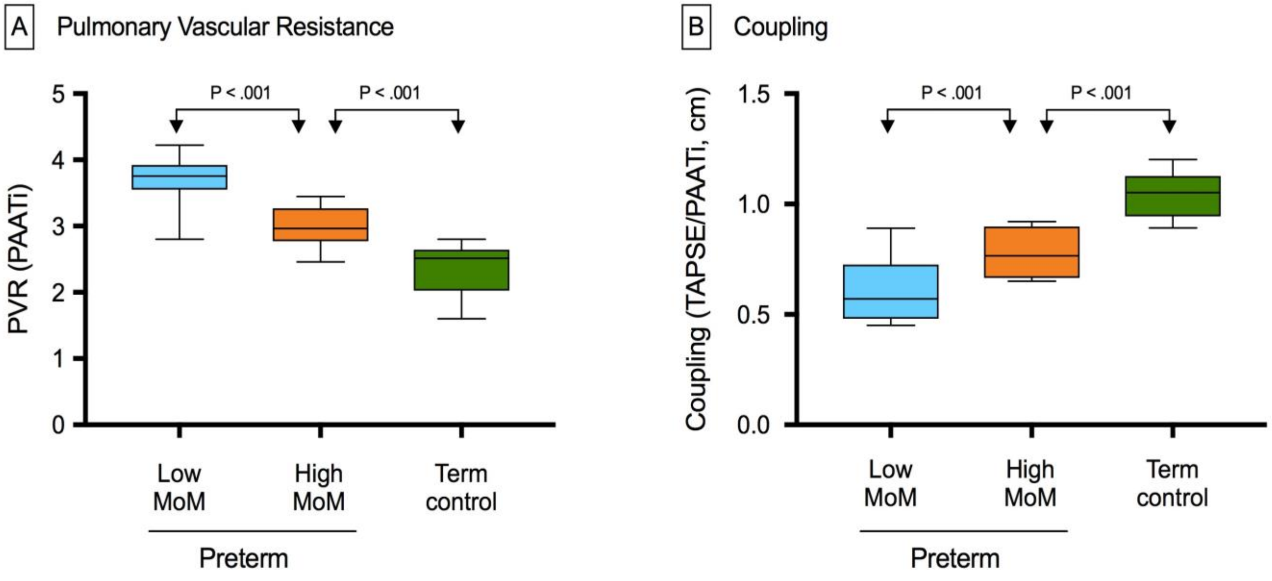

eFigure 5. At 1 year of age, preterm-born infants in the high mother's own milk (MoM) exposure group (orange) had lower pulmonary vascular resistance, PVR (A) - as measured by indexed pulmonary artery acceleration time (PAATi), and higher RV function to afterload coupling (B) - as measured by the ratio of tricuspid annular plane systolic excursion to PAATi compared with preterm-born infants in the low MoM exposure group (blue). Term-born control infants are shown in green. High MoM is defined as > 28 days of MoM exposure

© 2021 El-Khuffash A et al. *JAMA Network Open.*

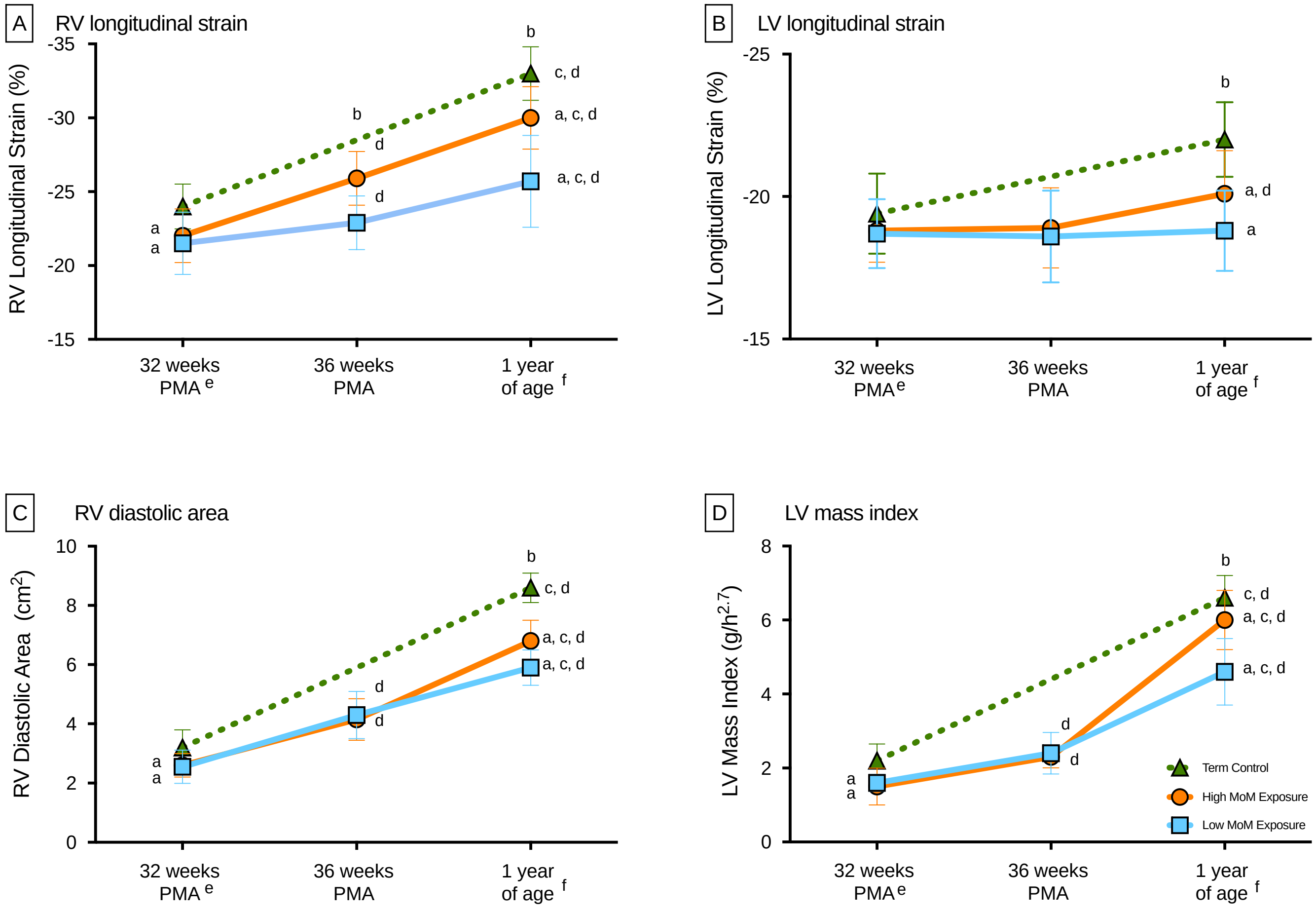

eFigure 6. Comparison of maturational patterns of right and left ventricular strain (A and B), right ventricular diastolic area (C), and left ventricular mass index, LMVI, g/h$^{2.7}$, (D) between preterm-born infants in the high mother's own milk (MoM) exposure group (orange) and the preterm-born infants in the low MoM exposure group (blue) over the first year of age. Term-born control infants are shown in green at 1 year of age. [a] P < .01 vs. term-born controls; [b] P < .01 between the high (n=42) and low (n=38) MoM exposure groups at each time point (with Bonferroni adjustment). [c] P < 0.01 change over time within the same group (ANOVA). [d] P < .01 compared with baseline of 32 weeks PMA [e] The first echocardiogram in the preterm-born infants was acquired at 32 weeks PMA at a median of 38 days (interquartile range of 33 to 45 days). Term infants had their first echocardiogram at a median of 32 days (interquartile range of 31 to 35 days). [f] At 1 year corrected age, the corrected age in days for all preterm infants was a median of 381 (355,324). The term infants received the 1-year of age echocardiogram at a median of 378 days (interquartile range of 365 to 399 days). High MoM is defined as > 28 days of MoM exposure

© 2021 El-Khuffash A et al. *JAMA Network Open.*

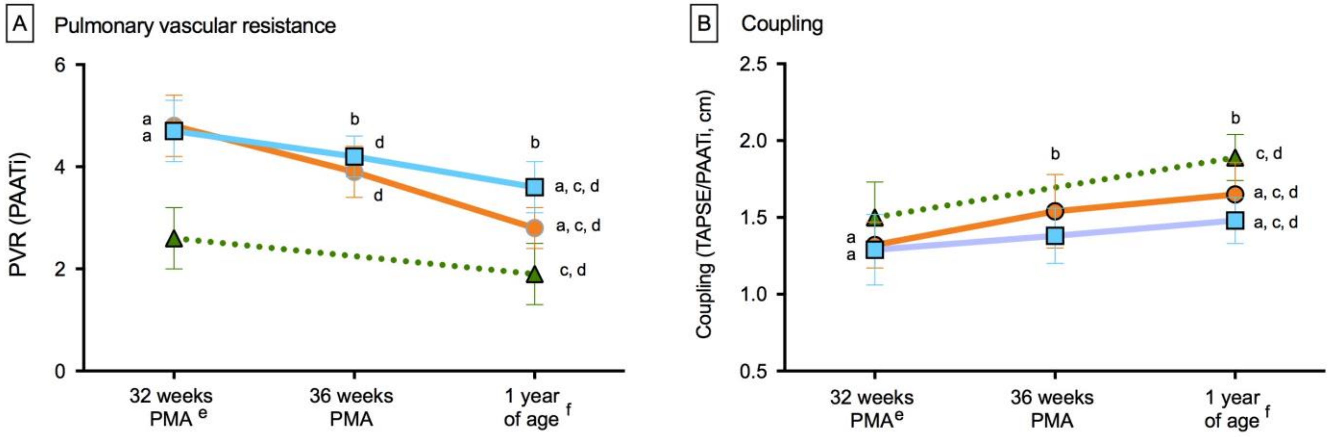

eFigure 7. Comparison of maturational patterns of pulmonary vascular resistance, PVR (A) - as measured by indexed pulmonary artery acceleration time (PAATi), and couplings (B) - as measured by the ratio of tricuspid annular plane systolic excursion to PAATi between preterm-born infants in the high MoM exposure group (orange) and the low MoM exposure group (blue) over the first year of age. Term-born control infants are shown in green. [a] P < .01 vs. term-born controls; [b] P < 0.01 between the high (n=42) and low (n=38) MoM exposure groups at each time point (with Bonferroni adjustment). [c] P < .01 change over within the same group (ANOVA). [d] P < .01 compared with baseline of 32 weeks PMA. [e] The first echocardiogram in the preterm-born infants was acquired at 32 weeks PMA at a median of 38 days (interquartile range of 33 to 45 days). Term infants had their first echocardiogram at a median of 32 days (interquartile range of 31 to 35 days). [f] At 1 year corrected age, the corrected age in days for all preterm infants was a median of 381 (355,324). The term infants received the 1-year of age echocardiogram at a median of 378 days (interquartile range of 365 to 399 days). High MoM is defined as > 28 days of MoM exposure

© 2021 El-Khuffash A et al. *JAMA Network Open.*

eReferences

1. Levy PT, Dioneda B, Holland MR, et al. Right Ventricular Function in Preterm and Term Neonates: Reference Values for Right Ventricle Areas and Fractional Area of Change. J Am Soc Echocardiogr. 2015;28(5):559-69.
2. Levy PT, Holland MR, Sekarski TJ, Hamvas A, Singh GK. Feasibility and reproducibility of systolic right ventricular strain measurement by speckle-tracking echocardiography in premature infants. Am Soc Echocardiogr. 2013 Oct;26(10):1201-1213.
3. Erickson CT, Patel MD, Choudhry S, et al. Persistence of right ventricular dysfunction and altered morphometry in asymptomatic preterm Infants through one year of age: Cardiac phenotype of prematurity. Cardiol Young. 2019;29(7):945-953.
4. Levy PT, Patel MD, Choudhry S, Hamvas A, Singh GK. Evidence of Echocardiographic Markers of Pulmonary Vascular Disease in Asymptomatic Preterm Infants at One year of Age. J Pediatr. 2018;197:48-56.
5. Pryhuber GS, Maitre NL, Ballard RA, et al. Prematurity and respiratory outcomes program (PROP): study protocol of a prospective multicenter study of respiratory outcomes of preterm infants in the United States. BMC Pediatr. 2015;15:37.
6. Poindexter BB, Feng R, Schmidt B, et al. Comparisons and Limitations of Current Definitions of Bronchopulmonary Dysplasia for the Prematurity and Respiratory Outcomes Program. Ann Am Thorac Soc. 2015;12(12):1822-1830.
7. Crispi F, Crovetto F, Gratacós E. Intrauterine growth restriction and later cardiovascular function. *Early Hum Dev.* 2018;126:23-27.
8. Keller RL, Feng R, DeMauro SB, et al. Bronchopulmonary Dysplasia and Perinatal Characteristics Predict 1-Year Respiratory Outcomes in Newborns Born at Extremely Low Gestational Age: A Prospective Cohort Study. J Pediatr 2017; 187:89-97.e83.
9. Sanchez AA, Levy PT, Sekarski TJ, Hamvas A, Holland MR, Singh GK. Effects of frame rate on two-dimensional speckle tracking-derived measurements of myocardial deformation in premature infants. Echocardiography. 2015;32:839-847.
10. Mitchell C, Rahko PS, Blauwet LA, et al. Guidelines for Performing a Comprehensive Transthoracic Echocardiographic Examination in Adults: Recommendations from the American Society of Echocardiography. J Am Soc Echocardiogr. 2019;32(1):1-64.
11. Lang RM, Badano LP, Mor-Avi V, et al. Recommendations for Cardiac Chamber Quantification by Echocardiography in Adults: An Update from the American Society of Echocardiography and the European Association of Cardiovascular Imaging. J Am Soc Echocardiogr. 2015;28(1):1-39.
12. Breatnach CR, Levy PT, James AT, Franklin O, El-Khuffash A. Novel Echocardiography Methods in the Functional Assessment of the Newborn Heart. Neonatology 2016:248-260.
13. Levy PT, El-Khuffash A, Patel M, et al. Maturational patterns of systolic ventricular deformation mechanics by two-dimensional speckle tracking echocardiography in preterm infants over the first year of age. J Am Soc Echocardiogr. 2017;30(7):685-698.
14. Patel M, Breatnach CR, James AT, et al. Echocardiographic Assessment of Right Ventricle Afterload in Preterm Infants: Maturational Patterns of Pulmonary Artery Acceleration Time Over the First Year of Age and Implications for Pulmonary Hypertension. J Am Soc Echocardiogr. 2019;32:884-894.
15. Choudhry S, Salter A, Cunningham TW, et al. Risk factors and prognostic significance of altered left ventricular geometry in preterm infants. J Perinatol. 2018;38(5):543-549.
16. Voigt J-U, Pedrizzetti G, Lysyansky P, et al. Definitions for a Common Standard for 2D Speckle Tracking Echocardiography: Consensus Document of the EACVI/ASE/Industry Task Force to Standardize Deformation Imaging. J Am Soc Echocardiogr. 2015;28(2):183-93.
17. Devereux RB, Reichek N. Echocardiographic determination of left ventricular mass in man. Anatomic validation of the method. Circulation. 1977;55(4):613-618.

© 2021 El-Khuffash A et al. *JAMA Network Open.*

18. Foster BJ, Mackie AS, Mitsnefes M, Ali H, Mamber S, Colan SD. A Novel Method of Expressing Left Ventricular Mass Relative to Body Size in Children. Circulation. 2008 May 27;117(21):2769-2775.
19. Choudhry S, Salter A, Cunningham T, et al. Normative Left Ventricular M-mode Echocardiographic Values in Preterm Infants Up To Two Kilograms. J Am Soc Echocardiogr. 2017;30(8):781-789.e4.
20. Haycock GB, Schwartz GJ, Wisotsky DH. Geometric method for measuring body surface area: a height-weight formula validated in infants, children, and adults. J Pediatr. 1978;93(1):62-6
21. Biton Y, Goldenberg I, Kutyifa V, et al. Relative Wall Thickness and the Risk for Ventricular Tachyarrhythmias in Patients With Left Ventricular Dysfunction. J Am Coll Cardiol. 2016;67(3):303-312.
22. Bussmann N, El-Khuffash A. Future perspectives on the use of deformation analysis to identify the underlying pathophysiological basis for cardiovascular compromise in neonates. Pediatr Res. 2019;85(5):591-595.
23. James AT, Corcoran JD, Franklin O, EL-Khuffash AF. Clinical utility of right ventricular fractional area change in preterm infants. Early Hum Dev. 2016 Jan;92:19-23
24. Levy PT, Patel MD, Groh G, et al. Pulmonary Artery Acceleration Time Provides a Reliable Estimate of Invasive Pulmonary Hemodynamics in Children. J Am Soc Echocardiogr. 2016;29(11):1056-1065.
25. Koestenberger M, Grangl G, Avian A, et al. Normal Reference Values and z Scores of the Pulmonary Artery Acceleration Time in Children and Its Importance for the Assessment of Pulmonary Hypertension. Circ Cardiovasc Imaging. 2017;10(1):e005336.
26. Vonk-Noordegraaf A, Westerhof BE, Westerhof N. The Relationship Between the Right Ventricle and its Load in Pulmonary Hypertension. J Am Coll Cardiol. 2017;69(2):236-243.
27. Jone PN, Schafer M, Pan Z, Bremen C, Ivy DD. 3D echocardiographic evaluation of right ventricular function and strain: a prognostic study in paediatric pulmonary hypertension. Eur Heart J Cardiovasc Imaging. 2018;19(9):1026-1033.
28. Levy PT, El-Khuffash A, Woo KV, Hauck A, Hamvas A, Singh GK. A Novel Noninvasive Index to Characterize Right Ventricle Pulmonary Arterial Vascular Coupling in Children. JACC Cardiovasc Imaging. 2019;12(4):761-763.
29. Levy PT, El Khuffash A, Woo KV, Singh GK. Right Ventricular-Pulmonary Vascular Interactions: An Emerging Role for Pulmonary Artery Acceleration Time by Echocardiography in Adults and Children. J Am Soc Echocardiogr. 2018;31(8):962-964.
30. Papile LA, Burstein J, Burstein R, Koffler H. Incidence and evolution of subependymal and intraventricular hemorrhage: a study of infants with birth weights less than 1,500 gm. J Pediatr. 1978 Apr;92(4):529-534.
31. Wang Y, Briere C-E, Xu W, Cong X. Factors Affecting Breastfeeding Outcomes at Six Months in Preterm Infants. J Hum Lact 2019; 35:80-89.

© 2021 El-Khuffash A et al. *JAMA Network Open.*
